# Host heterogeneity shapes clonal selection and organotropism in an extranodal lymphoma mouse model

**DOI:** 10.1038/s41419-026-09014-3

**Published:** 2026-07-10

**Authors:** Xiaoxi Li, Minyao Deng, Yong Jiang, Lingli Luo, Chenxiao Zhang, Hua Tang, Zhuang Wei, Jiayan Leng, Chuan Liu

**Affiliations:** 1 Department of Laboratory Medicine, School of Clinical Medicine & Laboratory Medicine, Jiangsu University, Zhenjiang, China; 2 State Key Laboratory of Cell Biology, Shanghai Institute of Biochemistry and Cell Biology, Center for Excellence in Molecular Cell Science, Chinese Academy of Sciences, University of Chinese Academy of Sciences, Shanghai, China; 3 Innovation Center for PreTrial Models and Premier Oncology, MD BioMedTech Co., Ltd., Shanghai, China; 4 Department of Hematology, The Affiliated People’s Hospital of Jiangsu University, Zhenjiang, China; 5 Department of Oncology, Shanghai Pudong Hospital, Fudan University Pudong Medical Center, Shanghai, China

**Keywords:** B-cell lymphoma, Cancer models

## Abstract

Tumor metastasis is a leading cause of patient mortality, with tumor dissemination and organotropism representing critical yet complex aspects of tumor biology that involve clonal selection and evolutionary dynamics. Current experimental models inadequately recapitulate the spatiotemporal dissemination and phenotypic heterogeneity of human cancers, highlighting a critical need for more physiologically relevant preclinical platforms. In this study, we utilized a kidney-tropic primary extranodal lymphoma model to explore the role of EFNB1 in lymphoma dissemination across two distinct mouse strains, C57BL/6J and C57BL/10J. Intriguingly, EFNB1-overexpressing lymphoma cells displayed markedly different progression rates and colonization patterns in these strains. EFNB1-null and EFNB1-high lymphomas derived from the two strains underwent distinct clonal selection and exhibited significant transcriptomic heterogeneity, including RNA base substitutions, as well as differential expression of nuclear histone genes and mitochondrial respiratory chain genes. Notably, clonal origin alone did not determine lymphoma organotropism. Collectively, this study establishes a readily reproducible extranodal lymphoma model and demonstrates that host heterogeneity can reshape transcriptional heterogeneity, drive tumor clonal selection, and ultimately dictate organotropism. These findings suggest that host heterogeneity may serve as a valuable prognostic indicator for lymphoma.

## Introduction

Extranodal lymphoma refers to lymphoma originating in tissues or organs outside the lymphatic system. Among B-cell lymphomas, diffuse large B-cell lymphoma (DLBCL) is the most common subtype, with approximately 40% of patients presenting extranodal involvement—a feature strongly associated with poor prognosis [1, 2]. Common sites of extranodal lymphoma in DLBCL include the gastrointestinal tract and skin. Transcriptomic and genomic studies indicate that extranodal DLBCL predominantly exhibits transcriptional profiles of the activated B-cell (ABC) subtype, with genetic variations frequently characterized by MYD88 and CD79B mutations, consistent with the MCD subtype [3]. Additionally, ABC/MCD DLBCL is associated with recurrent inactivating mutations in genes such as TBL1XR1 and BTG1, which are implicated in lymphoma dissemination [4, 5]. The International Extranodal Lymphoma Study Group (IELSG), a non-profit organization, is dedicated to advancing the understanding of extranodal lymphoma biology and improving treatment outcomes. Over the years, the IELSG has initiated numerous prospective clinical trials and retrospective studies to address the unique management challenges posed by various extranodal presentations (https://ielsg.org/). However, the limited availability of clinical samples and the high degree of genetic heterogeneity significantly constrain the ability of these trials to elucidate the biology of extranodal lymphoma and enhance therapeutic strategies. Despite the critical importance of understanding lymphoma organotropism for diagnosis, treatment, and prognosis, progress in this area remains hindered by the lack of robust experimental models.

Mouse cell-line-derived transplant models, also known as cell-line-derived allografts (CellDAs), involve the transplantation of mouse tumor cell lines into syngeneic immunocompetent mouse hosts [6]. A well-established CellDAs model for studying tumor dissemination is the orthotopic transplantation of 4T1 cells, which are known to metastasize to multiple organs, including the lungs, brain, intestines, and bones, in a seemingly random pattern. However, recent studies have demonstrated that non-genetic factors, such as obesity, can significantly influence dissemination patterns [7, 8]. These findings underscore that tumor dissemination is not entirely random and can be shaped by complex, often difficult-to-quantify factors, which may also affect organotropism. The *Eμ-Myc;Cdkn2a*^*−/−*^ cell line, derived from lymphomas in genetically engineered mouse models (GEMMs), is a widely used murine lymphoma cell line. When transplanted into recipient mice, it forms palpable tumors in multiple lymph nodes [9]. Recently, we identified a novel cell line, named MA, which spontaneously evolved from the *Eμ-Myc;Cdkn2a*^*−/−*^ cell line through transcriptional reprogramming. This MA cell line uniquely develops kidney-tropic extranodal lymphoma in the CellDAs model [10]. However, since the kidney is not a typical dissemination site for extranodal B-cell lymphoma [11], the applicability of th×e MA primary renal lymphoma model remains limited.

The malignancy of a tumor should be assessed based on multiple properties, including cellular proliferation, clonality, experimental transplantability, metastasis in solid tumors, and dissemination in hematologic neoplasms. EFNB1, a marker for germinal center B cells, plays a critical role in mediating interactions with T cells [12, 13]. Our previous studies have demonstrated that EFNB1 levels are significantly elevated in UTX-knockout lymphoma, and ectopic expression of EFNB1 enhances lymphoma dissemination [14]. EFNB1 expression levels are closely associated with drug response, cell origin, and prognosis in B-cell malignancies [15]. Furthermore, EFNB1 has been identified as a potential therapeutic target for B-cell lymphoma [16]. Collectively, these findings suggest that EFNB1 may play a key role in regulating lymphoma organotropism. To develop more clinically relevant extranodal lymphoma models, we generated MA cells stably overexpressing EFNB1, designated as MAB1. To further investigate the influence of host heterogeneity on lymphoma dissemination, we selected two mouse strains, C57BL/6J and C57BL/10J, as recipients for the CellDAs model. This study aims to provide new insights into the biology of lymphoma dissemination and to establish tractable preclinical mouse models of extranodal lymphoma.

## Materials and methods

### Cell lines

Murine B-cell lymphoma cell line *Eμ-myc;Cdkn2a*^*−/−*^ was a kind gift from Prof. Hai Jiang at the Center for Excellence in Molecular Cell Science. The MA cell line, originating from the *Eμ-Myc;Cdkn2a*^*−/−*^ cell line, was characterized in a recent study [10]. The MA cell line and MA cell strains were cultured in 45%DMEM, 45%IMDM, 10% fetal bovine serum, supplemented with 100 U/ml penicillin and streptomycin, 25 μM β-mercaptoethanol. The carefully regulated incubation environment maintained a consistent 5% CO_2_-saturated humidity and a stable temperature of 37 °C to facilitate optimal cell growth and proliferation. *Eμ-myc;Cdkn2a*^*−/−*^ cell line is derived from female mice according to RNA-seq data. A polymerase chain reaction (PCR)-based test for mycoplasma contamination was performed every 2 weeks.

### The establishment of lymphoma-derived cell strains

Efnb1 was amplified from murine cDNA and cloned into the retroviral vector MLS (LTR-MCS-SV40-GFP) in a previous study [14]. The MA cell line overexpressing Efnb1, designated as MAB1 in this study, was subsequently sorted by fluorescence-activated cell sorting (FACS) with the FACSAria II system (BD).

The lymphoma tissue, devoid of any visible disseminated organ, was finely ground using ground glass slides. Subsequently, the lymphoma cell suspension was incubated with a red blood cell lysis buffer to eliminate red blood cells and filtered to remove any cohesive impurities. The purity of lymphoma cells in the suspension derived from primary lymphoma tissue consistently exceeds 95%. After purification, the lymphoma cells were cultured in vitro and their progress monitored by flow cytometry. Cell morphology, encompassing size and granularity, was evaluated using forward scatter (FSC) and side scatter (SSC), respectively. Over a period of 3 weeks, cell strains exhibiting stable cell morphology were preserved as lymphoma-derived cell strains. All flow cytometry analyses were conducted using the CytoFlex instrument (Beckman), and the data were thoroughly analyzed utilizing CytExpert software (Beckman).

The parental MA and MAB1 cell lines, as well as the MA/MAB1 lymphoma-derived cell strains, were cultured in B-cell medium, which comprised of a mixture of 45%DMEM, 45%IMDM, 10% fetal bovine serum. The medium was further supplemented with 100 U/ml penicillin and streptomycin, along with 25 μM β-mercaptoethanol. Additionally, a PCR-based test for mycoplasma contamination was conducted every 2 weeks.

### Syngeneic lymphoma reconstruction model

Given that the MA cell line utilized in this study was originally derived from female mice and failed to form tumors upon transplantation into male mice, all recipient mice employed in this research were of the female gender.

The syngeneic lymphoma reconstruction model utilized in this study belongs to a category of mouse cell-line-derived transplant models, commonly referred to as CellDAs. For the establishment of this model, 10^6^ cells suspended in 200 μl of Dulbecco’s Phosphate Buffered Saline (DPBS) were injected into recipient mice aged between 6 and 8 weeks, specifically through the tail vein (intravenously, IV). The observation of moribund behavior commenced 3 weeks post-transplantation, and by the fourth week, typical symptoms of lymphoma progression manifested. These symptoms encompassed a hunched back, dull hair coat, retardation in movement, and an abdominal bulge. Recipient mice were closely monitored for the emergence of any of these symptoms, and once detected, they were euthanized for the evaluation of lymphoma dissemination.

The recipient mice of the C57BL/6J and C57BL/10J strains in the comparative experiment were purchased from GemPharmatech (Nanjing, CN), named as C57BL/6JGpt and C57BL/10JGpt, respectively. A sample size of five mice per group was chosen based on the following considerations. First, preliminary experiments suggested that five mice per group were sufficient to consistently observe the phenotypic differences of interest, with acceptable variability. Second, this sample size aligns with the principle of the 3Rs (Replacement, Reduction, Refinement) by minimizing the number of animals while still enabling a reliable interpretation of the results. Third, given the considerable inter-individual variability in disease onset and progression in this extranodal lymphoma model, a larger cohort (e.g., >5 per group) would have substantially increased the complexity of simultaneous dissection, sample collection, and data recording, thereby compromising the feasibility of the experiment. The recipient mice of the C57BL/6J strain in other experiments were purchased from the Laboratory Animal Research Center in Jiangsu University. For mice purchased from commercial companies, we housed them in a new facility for more than 2 weeks before conducting experiments. All recipient mice in this study are female. The detailed information of the lymphoma reconstitution experiments is listed in Supplementary Table 1. Animals were randomly allocated to each group using a random number table. The investigator was blinded to group allocation during data collection and analysis.

All mice were housed in a specific pathogen-free environment at the Laboratory Animal Research Center in Jiangsu University and treated in strict accordance with protocols, which were approved by the Animal Care and Use Committee of the Laboratory Animal Research Center, Jiangsu University.

### RNA and DNA isolation and reverse transcription

2 × 10^6^ cells of various cell lines/strains and purified lymphoma cells were preserved in Trizol at −80 °C for RNA extraction and at −20 °C for DNA extraction. Total RNA was extracted using Trizol reagent kit (15596018, Invitrogen) according to the manufacturer’s protocol. Genomic DNA (gDNA) was extracted using FastPure Cell/Tissue DNA Isolation Mini Kit (DC102, Vazyme) according to the manufacturer’s protocol. Complementary DNA (cDNA) was produced by reverse transcription of total RNA using HiScript III 1st Strand cDNA Synthesis Kit (R312, Vazyme) according to the manufacturer’s protocol.

### DNA methylation analysis

The gDNA was converted using the EpiArt DNA Methylation Bisulfite Kit (EM101, Vazyme), following the manufacturer’s recommended protocol. Subsequently, the bisulfite-converted gDNA was amplified using the EpiArt HS Taq Master Mix (EM201, Vazyme). MethPrimer1 was designed to amplify both endogenous and exogenous Efnb1, whereas MethPrimer2 is capable of amplifying only exogenous Efnb1. All primers used for DNA methylation analysis are listed as follows.

MethPrimer1-Forward: 5′-AGTTTTAAAGGGGGTAGTGGTATAG-3′.

MethPrimer1-Reverse: 5′-TAACTAACACACATTCCACAAAATC-3′.

MethPrimer2-Forward: 5′-GGAAGAGAAGAGTGGTTTAGGTGTA-3′.

MethPrimer2-Reverse: 5′-CCACTACCCCCTTTAAAACTAACTAA-3′.

The resulting amplicons were cloned into TA-TOPO vectors (C601, Vazyme), and positive clones were selected for bisulfite sequencing. For DNA methylation analysis, primers were designed using MethPrimer (http://www.urogene.org/cgi-bin/methprimer/methprimer.cgi). The visualization and quality control of the DNA methylation data obtained from bisulfite sequencing were meticulously carried out using the BiQ Analyzer software.

### Transcriptome Bulk-sequencing

The total RNA samples were transported on dry ice to Gene Denovo Biotechnology Company (Guangzhou, China) for library construction and bulk-sequencing. Two technical replicates were employed for each sample of MA and MAB1 lymphoma, as well as the MAB1-1/2/3 cell strains. Prior to library construction, the RNA quality was evaluated using an Agilent 2100 Bioanalyzer (Agilent Technologies).

The subsequent library construction process was described as follows. The mRNA enriched by Oligo(dT) beads was fragmented into short fragments using the fragmentation buffer and reverse transcribed into cDNA by using NEBNext Ultra RNA Library Prep Kit for Illumina (#7530, New England Biolabs). The purified double-stranded cDNA fragments were end-repaired, A base added, and ligated to Illumina sequencing adapters. The ligation reaction was purified with the AMPure XP Beads (1.0X). Ligated fragments were subjected to size selection by agarose gel electrophoresis and PCR amplified. The resulting cDNA library was sequenced using Illumina Novaseq6000.

### Identification of SNVs and RNA editing

The GATK [17] (version 3.4-46) was used for calling variants of transcripts, and ANNOVAR was used for SNP/InDel annotation. The function, genome site, and type of variation of SNPs were also analyzed.

To screen reliable editing sites from single-nucleotide variations (SNVs), the following criteria were applied: Removing the low-quality SNVs while calling SNVs by GATK; Correcting the SNVs around the InDel region; Choosing non-overlapping SNVs in the UTR and exon region; Choosing SNVs with reference reads ≥2, and variate reads ≥3; Choosing SNVs with the mutation frequency between 0.1 and 0.9. 441252 SNVs were identified in all 26 samples.

The mutational signature of human lymphoma was acquired from the MutaGene database [18].

### Sanger sequencing verification

To distinguish DNA mutation and RNA editing, both gDNA and cDNA were analyzed for SNVs. Primer pairs for SNVs of DNA mutation were designed in the introns flanking the SNV sites. Primer pairs for SNVs of RNA editing were designed in exons flanking the SNV sites. Amplicons were obtained using Platinum HS PCR Master Mix (Invitrogen) and directly sequenced by Sanger sequencing to determine the mutation frequency or RNA editing efficiency. All primers for SNV verification were listed in Supplementary Table 2.

### Immunoglobulin gene (IG) rearrangement analysis

Seven genes of Ighv and Igkv (Ighv2-2, Ighv2-3, Ighv5-2, Igkv3-1, Igkv4-70, Igkv13-85, Igkv19-93) were used for IG rearrangement analysis. To analyze the rearranged Ig genes, gDNA was used as the template, and the forward and reverse primers of Ig genes were designed in intron 1–2 and exon 2, respectively. Amplicons (300 bp to 400 bp) were obtained using Platinum HS PCR Master Mix (Invitrogen) and identified by agarose gel electrophoresis. All primers for clonal rearrangement analysis of Ig genes were listed in Supplementary Table 2.

### Differentially expressed genes (DEGs) analysis

The mapped reads of each sample were assembled by using StringTie v1.3.1 in a reference-based approach. For each transcription region, an FPKM (fragment per kilobase of transcript per million mapped reads) value was calculated to quantify its expression abundance and variations, using RSEM software. RNA differential expression analysis was performed by the DESeq2 software between two different groups. The genes with the parameter of false discovery rate below 0.05 and absolute fold change ≥2 were considered differentially expressed genes (DEGs).

Principal component analysis (PCA) was performed with the R package gmodels (http://www.r-project.org/) in this experience. PCA is a statistical procedure that converts hundreds of thousands of correlated variables (gene expression) into a set of values of linearly uncorrelated variables called principal components. PCA is largely used to reveal the relationship of the samples.

### Pathway and interactome enrichment analysis

Pathway enrichment analysis and protein–protein interaction (PPI) enrichment of DEGs were performed with Metascape (https://metascape.org). Pathway enrichment analysis has been carried out with GO Biological Processes. All genes in the genome have been used as the enrichment background. Terms with a *p* value < 0.01, a minimum count of 3, and an enrichment factor >1.5 (the enrichment factor is the ratio between the observed counts and the counts expected by chance) are collected and grouped into clusters based on their membership similarities. More specifically, *p* values are calculated based on the cumulative hypergeometric distribution. PPI enrichment analysis has been carried out with the following databases: STRING and BioGrid. Only physical interactions in STRING (physical score >0.132) and BioGrid are used. The Molecular Complex Detection (MCODE) algorithm has been applied to identify densely connected network components.

### Trend analysis

Gene expression pattern analysis was used to cluster genes of similar expression patterns for multiple samples. To examine the expression pattern of DEGs, the expression data of each sample were normalized to 0, log2 (v1/v0), log2(v2/v0), and then clustered by Short Time-series Expression Miner software (STEM) [19]. The parameters were set as follows: (1) Maximum Unit Change in Model Profiles between Time Points is 1; (2) Maximum number of output profiles is 20, and similar profiles will be merged; (3) Minimum ratio of fold change of DEGs is no less than 2.0. The clustered profiles with *p* value ≤ 0.05 were considered as significant profiles.

### Prognostic analysis

Survival analysis was performed with the online program SurvExpress [20]. A human DLBCL dataset (Lenz Staudt Lymphoma GSE10846 [21], *n* = 414) was chosen for survival analysis. The prognostic index (PI) was calculated by the expression value and the Cox model to generate the risk groups. An optimization algorithm was applied in risk grouping. The SurvExpress program was performed according to the tutorial.

### Correlation analysis of gene expression

The RNA-seq data of DLBCL lymphoma (The GOYA trial GSE125966 [22], *n* = 553) were used for correlation analysis between the expression of EFNB1 and nuclear histone genes, as well as mitochondrial respiratory chain genes. Additionally, the RNA-seq data of 23 human DLBCL cell lines, acquired from Cell Model Passports [23], were employed to analyze the expression levels of cholesterol metabolism genes.

### ATP and NADH content measure

ATP content was measured using the Enhanced ATP assay kit (S0027, Beyotime) according to the manufacturer’s protocol. NADH was measured using NADH Assay Kit with WST-8 (S0175, Beyotime) according to the manufacturer’s protocol. Luminescence and absorbance were acquired by Cytation5 (BioTek) and analyzed using Gen5 software (BioTek).

### COO classification

The Cell-of-Origin (COO) score was derived from the expression levels of 20 genes associated with the ABC and germinal center B-cell (GCB) lymphoma subtypes. These genes were categorized into two panels: ABC-associated genes (*n* = 12): SH3BP5, IRF4, PIM1, ENTPD1, BLNK, CCND2, ETV6, FUT8, BMF, IL16, PTPN1. GCB-associated genes (*n* = 8): ITPKB, MME, BCL6, MYBL1, DENND3, NEK6, LMO2, LRMP, SERPINA9.

Gene expression values were processed as follows: Quantile normalization and log₂ transformation were applied to raw expression data. *Z*-score normalization was performed across all 20 genes. For each sample, the mean *z*-score was computed separately for the ABC and GCB gene panels to generate subtype-specific scores (ABC score and GCB score). The COO score was calculated as the difference between the ABC and GCB scores (COO = ABC score − GCB score).

Samples were classified into COO subtypes based on the following criteria: ABC subtype: COO score >0.25 and GCB score <0.75. GCB subtype: COO score <−0.25 and ABC score <0.75. Unclassified: Samples not meeting the above criteria.

### Statistical analysis

The statistical methods employed were tailored to the specific type of experiment, including unpaired *t*-tests, simple linear regression, and log-rank (Mantel–Cox) tests, as detailed in the respective figure legends. Statistical significance was determined by *P* values less than 0.05. Significance levels were denoted as follows: *P < 0.05, **P < 0.01, ***P < 0.001, and ****P < 0.0001. All statistical analyses were conducted using GraphPad Prism 9.0 software (GraphPad Software).

## Results

### EFNB1 facilitates the random colonization of lymphomas

**Fig. 1:**
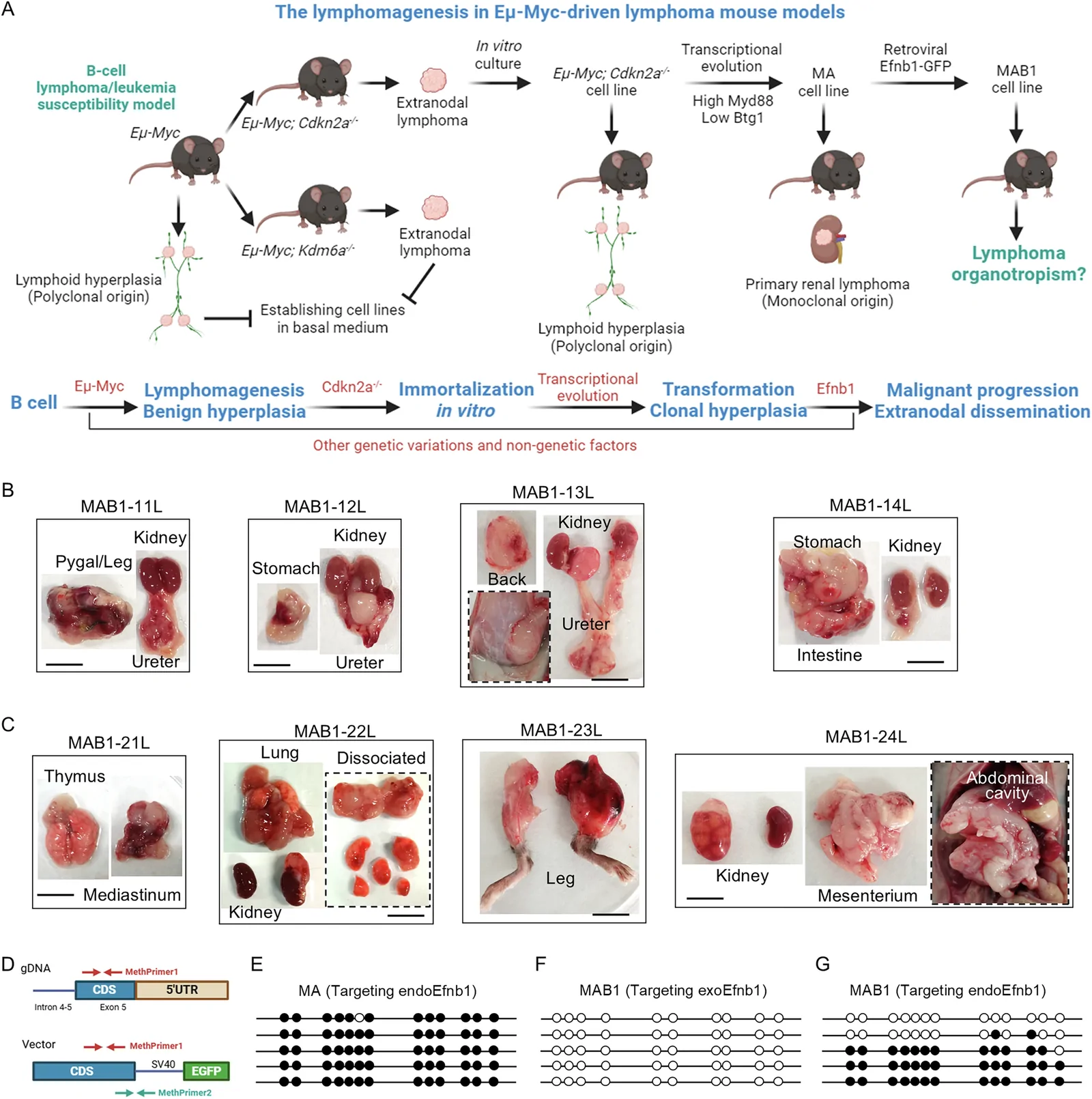
Overexpression of EFNB1 leads to random colonization of lymphoma cells. **A** Diagram of lymphoma progression and dissemination in multiple Eμ-Myc-driven lymphoma models, including GEMMs and CellDAs. **B**, **C** Representative images of extranodal colonization of MAB1 lymphoma in C57BL/6 recipient mice across two independent replicate experiments. Bar, 10 mm. The image within the dashed outline is not presented to scale. **D** Diagram of primer binding sites for methylation analysis of the Efnb1 gene. MethPrimer1 is designed within the EFNB1 gene, targeting both endogenous and exogenous EFNB1. MethPrimer2 is designed to span both the EFNB1 gene and the vector, targeting exogenous EFNB1. Methylation analysis of endogenous Efnb1 in MA cell line (**E**), exogenous Efnb1 in MAB1 cell line (**F**), and endogenous/exogenous Efnb1 in MAB1 cell line (**G**). Methylated cytosine, filled circles. Unmethylated cytosine, empty circles.

To date, despite the emergence of over 170 GEMMs in the past 40 years [24], there remains considerable controversy regarding whether the *Eμ-Myc* transgenic mouse model faithfully recapitulates human B-cell lymphoma [25]. Firstly, drawing from current knowledge and our experiences with *Eμ-Myc*-related GEMMs and CellDAs, we offer a brief elaboration on our comprehension of these models and their clinical relevance. Malignancies in B-cell lymphoma are widely considered a result of abnormal differentiation [26], with human Non-Hodgkin B-cell lymphoma (B-NHL) primarily originating from mature B cells [25]. Although the *Eμ-Myc* transgenic mice can develop enlarged lymph nodes in the inguinal, brachial, and cervical regions, as well as splenomegaly [27], most tumor cells are polyclonal and originate from the pre-B stage. Additionally, these cells often fail to survive and proliferate in basic culture media, characteristics that are more indicative of lymphoid hyperplasia rather than lymphoma (Fig. 1A). However, it is crucial to recognize that lymphomas derived from *Eμ-Myc* mice can exhibit significant heterogeneity [28] and can also originate from the mature B-cell stage [14]. Consistent with the two-hit theory [29], lymphoma development in *Eμ-Myc* mice frequently requires additional genetic alterations, including the inactivation of tumor suppressor genes like TP53, CDKN2A, and BCOR [30, 31]. Therefore, the Eμ-Myc transgenic mice cannot be considered a static, unambiguous, and singular lymphoma model. Moreover, the nature of the secondary hit has been reported to significantly impact lymphoma progression through its modulation of tumor immunosurveillance mechanisms [32]. Given the multistep nature of tumorigenesis, we proposed that the *Eμ-Myc* transgenic mouse model should be viewed as a susceptibility model for B-cell lymphoma/leukemia, with the pathological progression of lymphoma/leukemia being largely dependent on secondary hit (Fig. 1A).

Drawing from our comprehensive understanding of lymphomagenesis in GEMMs and CellDAs, we have delineated four distinct stages in the pathological progression of lymphoma: benign hyperplasia, immortalization, clonal hyperplasia (transformation), and dissemination (Fig. 1A). The scientific inquiry posed by this study centers on a pivotal question: whether overexpression of EFNB1 triggers the evolution of kidney-tropic lymphoma into clinically pertinent models of extranodal lymphoma. This exploration serves as a probe into the fascinating phenomenon of lymphoma organotropism, furthering our understanding of the malignancy progression in extranodal lymphomas.

To investigate the impact of EFNB1 on lymphoma organotropism, we stably overexpressed EFNB1 in the MA cell line, named MAB1. Unexpectedly, the lymphomas arising from CellDAs with the MAB1 cell line did not confine themselves to a specific anatomical location; instead, they exhibited a remarkable diversity and host heterogeneity (Fig. 1B). To ensure the reliability of our findings, we conducted additional independent experiments and observed similar outcomes (Fig. 1C). Among the 8 recipient mice from two independent experiments, the colonization sites of MAB1 lymphoma encompassed various bodily regions, including the stomach and leg (Fig. 1B, C), which are all common sites in human DLBCL. Notably, the kidney was no longer a major colonization site for MAB1 lymphoma (Fig. 1B, C), suggesting that while EFNB1 confers lymphoma cells with a random colonization capacity, it simultaneously abolishes their ability to colonize the kidney, their original organ tropism. These results not only underscore our successful establishment of a novel, clinically relevant primary extranodal lymphoma (PEL) model but also firmly establish EFNB1 as a pivotal regulatory gene governing lymphoma organotropism.

Human B-cell lymphomas predominantly arise from mature B cells, with EFNB1 expression first detected in germinal center (GC) B cells during their differentiation and selection processes. Therefore, elucidating the mechanism that downregulates EFNB1 expression during the early B-cell stage could provide valuable insights for targeting EFNB1 to halt tumor dissemination. Our previous study has demonstrated the absence of EFNB1 expression in several cell lines, including *Eμ-Myc;Cdkn2a*^*−/−*^, MA, as well as *Eμ-Myc* lymphomas arising from pro-B/pre-B cells. Conversely, lymphomas arising from mature B cells, such as *Eμ-Myc;Kdm6a*^*−/−*^, exhibit high EFNB1 expression [15]. Given that EFNB1 is located on the X chromosome, we hypothesize that DNA methylation might be the major mechanism regulating the silencing of EFNB1 expression. To test this hypothesis, we designed two primers targeting endogenous and exogenous EFNB1, respectively (Fig. 1D). By analyzing the DNA methylation status of the EFNB1 gene in both the MA and MAB1 cell lines, we found that the endogenous EFNB1 gene in MA cells is hypermethylated, while the exogenous EFNB1 gene in MAB1 cells is hypomethylated (Fig. 1E, F). Since the endogenous primer targets both endogenous and exogenous EFNB1 genes, both hypermethylated and hypomethylated EFNB1 genes are detectable in MAB1 cells (Fig. 1G). These findings suggest that inducing re-methylation of the EFNB1 gene as an approach to suppress its expression might represent a potential therapeutic strategy for preventing lymphoma dissemination.

In summary, these findings establish EFNB1 as a crucial regulatory gene that governs lymphoma organotropism and facilitates the random colonization of lymphomas. Moreover, they underscore the key role of non-genetic mechanisms, as opposed to genetic variations, in driving the malignant progression of lymphoma.

### Host heterogeneity influences lymphoma progression and dissemination

Tumorigenesis is an intricate process, influenced by both the inherent heterogeneity of the tumor itself and the diverse characteristics of individual patients. Factors such as age, physical condition, and immune status play crucial roles in determining tumor progression and prognosis. Nevertheless, the current tumor mouse models, which are typically developed in inbred strains, pose limitations in exploring and discussing the impact of host heterogeneity on tumor progression.

To explore the potential influence of intrinsic differences in recipient mice on lymphoma progression, we conducted a comparative analysis of the tumorigenic potential and organ tropism exhibited by MA and MAB1 lymphomas in two mouse strains, C57BL/6J and C57BL/10J, hereinafter referred to as B6 and B10. Given that B6 and B10 are closely related inbred substrains with shared genetic ancestry [33, 34], they provide an ideal model system for dissecting the impact of the host differences on lymphoma pathogenesis. While GFP overexpression can elicit immunogenic responses and alter tumor growth in some tumor models [35], such as the 4T1 breast cancer model (BALB/c background), our experiences confirm that retroviral GFP transduction does not affect lymphomagenesis in the MA lymphoma model (C57BL/6 background). The lymphoma dissemination pattern of the MA-EV model was similar to that of the MA model, primarily involving the kidneys without affecting other sites, and no statistically significant difference was observed between the two models (Supplementary Fig. 1A, B). Additionally, to exclude potential effects of random integration of retroviral vectors in clonally derived lymphomas, we used the parental, untransduced MA cell line as the control for MAB1, rather than the GFP-expressing MA-EV (Empty vector) cells.

**Fig. 2:**
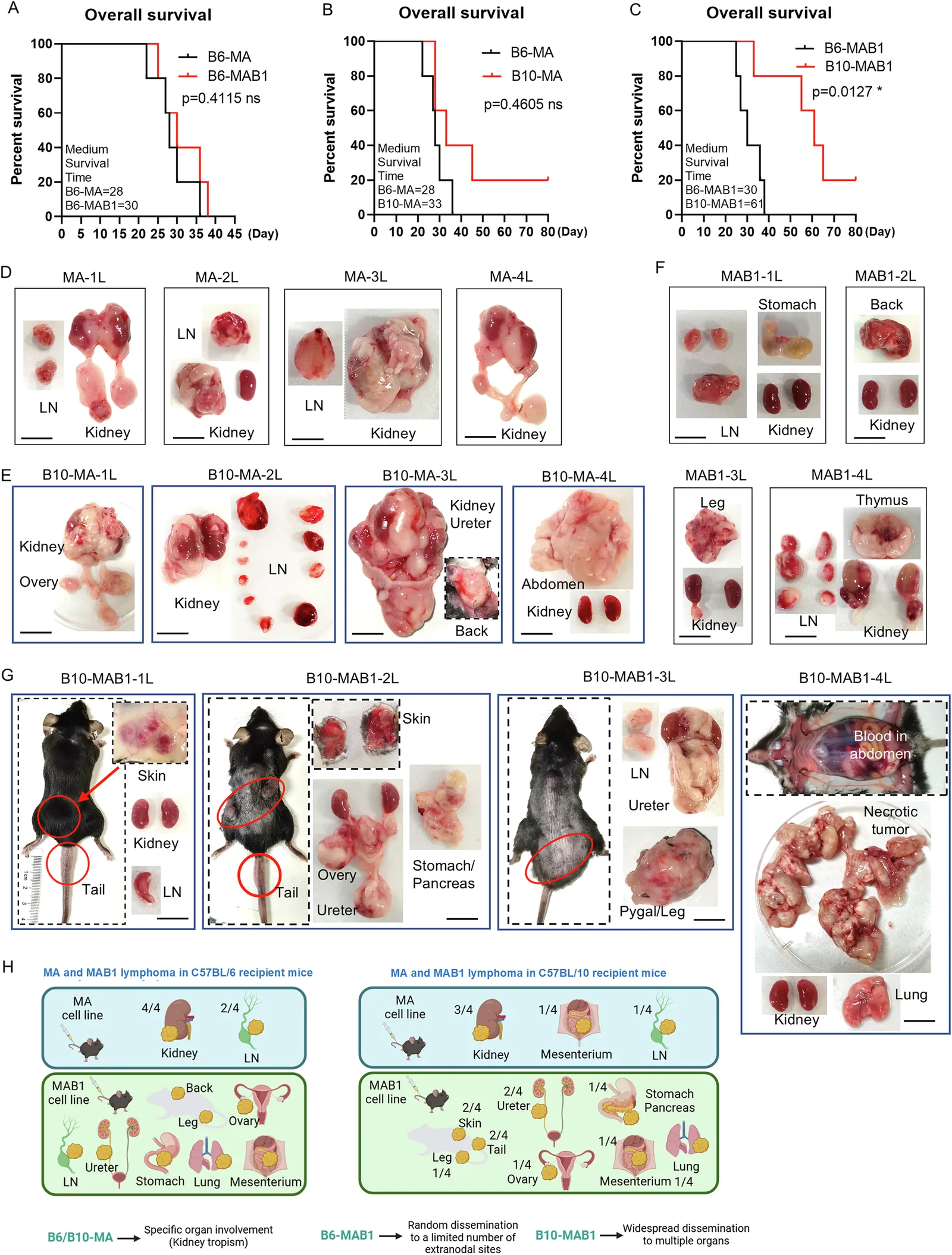
MAB1 cells exhibit distinct lymphoma progression and dissemination patterns in B6 and B10 recipient mice. **A** Survival curve of the B6-MA and B6-MAB1 mice. *n*(B6-MA) = 5, *n*(B6-MAB1) = 5. **B** Survival curve of the B6-MA and B10-MA mice. *n*(B6-MA) = 5, *n*(B10-MA) = 5. **C** Survival curve of the B6-MAB1 and B10-MAB1 mice. *n*(B6-MAB1) = 5, *n*(B10-MAB1) = 5. The log-rank (Mantel–Cox) test was used to evaluate the survival curves’ significance. Representative images of extranodal colonization of MA lymphoma in B6 (**D**) and B10 (**E**) recipient mice. Representative images of extranodal colonization of MAB1 lymphoma in B6 (**F**) and B10 (**G**) recipient mice. **H** Extranodal dissemination characteristics of MA and MAB1 lymphomas in B6 and B10 recipient mice. Bar, 10 mm. The image within the dashed outline is not presented to scale.

Our results revealed that both MA and MAB1 cells were capable of inducing tumor formation in B10 mice. The endpoint of our CellDAs experiments was determined by the onset of severe morbidity or the development of significant tumors in the recipient mice. Notably, we did not observe any significant difference in the overall survival time between MA and MAB1 lymphoma-bearing B6 mice (Fig. 2A, Supplementary Fig. 1C). Similarly, there was no significant difference in the overall survival time of MA lymphoma in B6 and B10 mice (Fig. 2B). However, unexpectedly, the overall survival time of MAB1 lymphoma-bearing B10 mice was significantly prolonged, approximately twice as long as that of B6 mice (Fig. 2C). This finding suggests a corresponding extension in the time required for lymphoma formation in B10 mice, highlighting the potential influence of recipient mouse strain on lymphoma progression.

Regarding lymphoma organotropism, MA lymphoma primarily colonized the kidneys in both B6 and B10 mice (Fig. 2D, E, Supplementary Fig. 1D). However, MAB1 lymphomas arising in B10 mice exhibited a more random pattern, involving multiple organs (Fig. 2F, G), and this pattern was significantly distinct from that observed in B6 mice. Unlike in B6 mice, MAB1 lymphomas in B10 mice exhibited a tendency to simultaneously invade multiple organs (Fig. 2G), presenting as a systemic lymphoma. We speculate that the slower progression of lymphoma might contribute to the observed multi-site colonization in MAB1 lymphoma-bearing B10 mice. Additionally, a noteworthy observation is the emergence of colonization sites in B10 mice that were absent in B6 mice, particularly the skin (Fig. 2G). These findings hint at a genetic predisposition in B10 mice towards developing both systemic and cutaneous lymphoma.

Regarding the occurrence of tail lymphomas in B10, we propose two plausible explanations. One is that dormant lymphoma cells may have remained in the tail following injection and could have been reawakened. The other is that minor injuries caused by the tail vein injection may favor the colonization of lymphoma cells [36]. However, given the lack of clinical significance associated with tail lymphomas, we refrained from further discussion.

Additionally, we conducted histological examinations of the most MAB1 lymphomas (Supplementary Fig. 2) and identified distinct histological features, including the “starry sky” pattern in EFNB1-overexpressing MAB1-2L and MAB1-4L lymphomas. The “starry sky” pattern is one of the hallmarks of aggressive B-cell lymphomas [37], though their pathobiological relevance remains poorly understood.

Notably, histopathological heterogeneity across species-specific lymphoma morphologies poses significant challenges for direct comparative analyses. Given the study’s focus on transcriptome-driven mechanisms of lymphomagenesis, we prioritized RNA-seq-based molecular stratification over histopathological comparisons, as this approach provides higher-resolution insights into subtype-specific transcriptional programs. While histopathological correlations are essential for clinical translation, this study emphasizes transcriptome-centric approaches to overcome interspecies barriers in lymphoma mouse models.

In summary, MA lymphomas displayed a distinct tropism, primarily colonizing the kidneys in both mouse strains. Conversely, MAB1 lymphomas exhibited multi-organ dissemination in B6 and B10 mice, mirroring the clinical observations. Notably, MAB1 lymphomas in B10 mice developed into systemic lymphoma and demonstrated a unique tropism towards the skin, indicating the genetic predisposition of B10 mice for lymphoma organotropism (Fig. 2H). We speculate that host heterogeneity may exert a lesser impact on benign or early-stage transformed tumors, while its influence is more profound in malignant or aggressive tumors. These results highlight that individual intrinsic differences can significantly influence lymphoma progression and organotropism, which is also dependent on the lymphoma itself. These findings provide novel experimental evidence supporting the correlation between individual intrinsic differences and tumor malignancy.

### EFNB1 influences tumor clonal selection with host heterogeneity

The intricate nature of tumor clonal selection and organotropism poses significant challenges in understanding their complexity. However, the monoclonal proliferative nature of lymphoma offers a more straightforward approach to analyzing clonal selection patterns, organotropism, as well as the adaptive transcriptional changes and their plasticity. Moreover, extrinsic factors encompass a complex array of variables—including murine immunological status, metabolic profiles, and dietary interventions—that pose significant challenges to systematic evaluation and experimental validation. We posit that lymphoma’s aggressive behavior remains intrinsically governed by lymphoma cell-autonomous mechanisms, even when external stimuli induce transcriptional reprogramming of dissemination-associated molecules such as integrins. Therefore, this study specifically focuses on the ultimate transcriptional consequences of environmental perturbations on lymphoma cells.

**Fig. 3:**
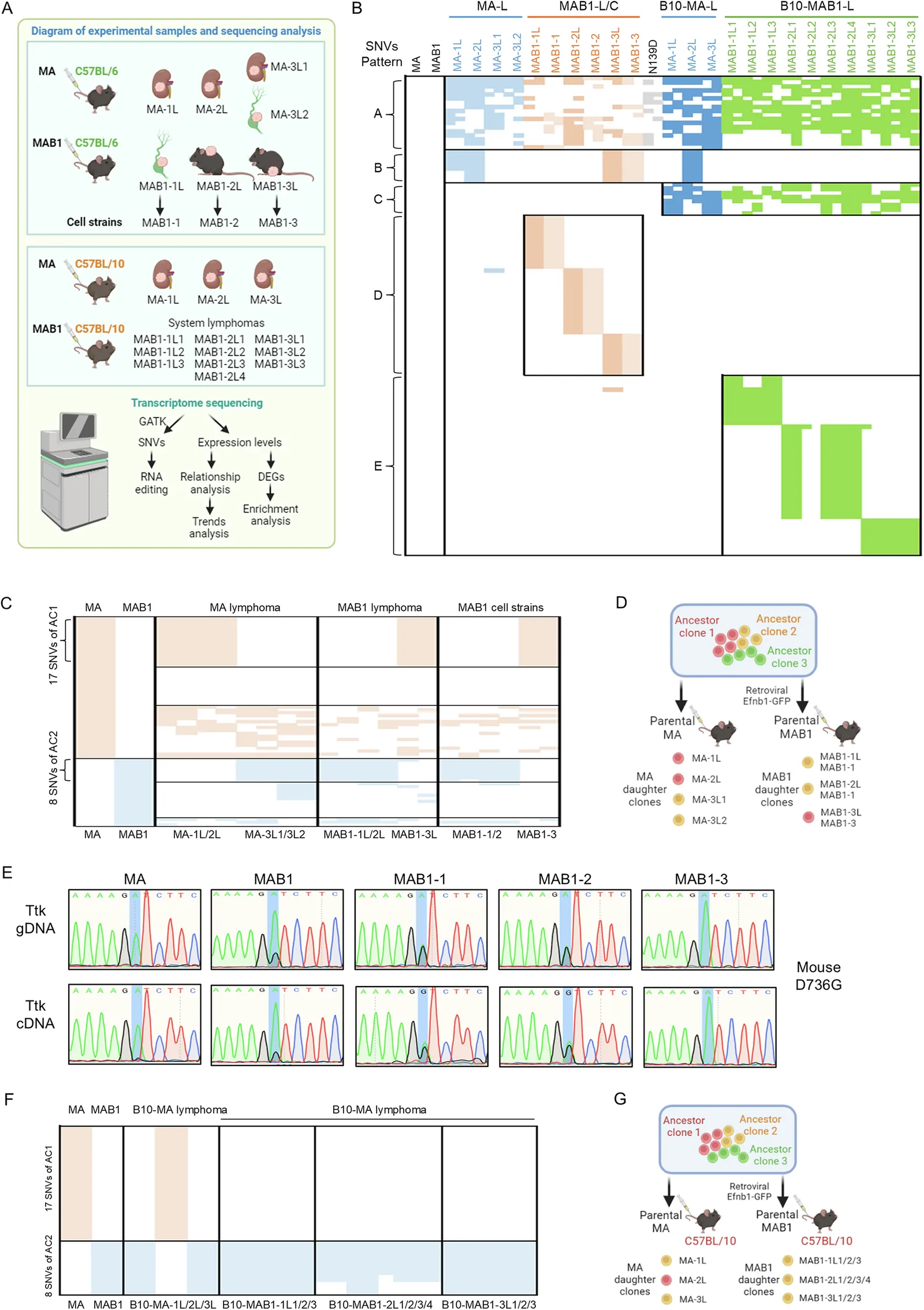
The clonal origin of MA and MAB1 lymphomas arising in B6 and B10 recipient mice. **A** Diagram of lymphoma samples and transcriptomic sequencing analysis. As indicated in the diagram, this study analyzed 20 lymphoma samples arising in 12 recipient mice, with three mice in each of the four experimental groups, along with 3 cell lines and 3 lymphoma-derived cell strains. **B** The heterozygous SNV status across 26 samples. SNVs with a quality score greater than 900 that are newly emerged in lymphoma samples but absent in the parental MA and MAB1 cell lines were selected for clonal origin analysis. Five SNV patterns, A–E, were identified as indicated. Colored regions represent heterozygous single-nucleotide variants (SNVs), while blank areas indicate wild-type homozygous loci. Colors denote distinct categories of lymphoma subtypes or cell lines. **C** The heterozygous SNV status of MA and MAB1 lymphomas arising from B6 recipient mice. Unique SNVs of the parental MA/MAB1 cell lines were selected for clonal origin analysis of B6-MA/MAB1 lymphomas. 17 MA-specific SNVs and 8 MAB1-specific SNVs that reappear in MA/MAB1 lymphomas are designated as the defining SNVs for ancestral clone 1/2(AC1/2). Colored regions represent heterozygous single-nucleotide variants (SNVs), while blank areas indicate wild-type homozygous loci. Distinct colors denote cell line-specific heterozygous single-nucleotide variants (SNVs) in MA (orange) and MAB1 (blue) cells, which were utilized as molecular markers to reconstruct ancestral clone divergence patterns. **D** The diagram of the clonal origin of B6-MA and B6-MAB1 lymphomas/cell strains. **E** Sequencing validation of the frequency of an SNV locus on the Ttk gene in MAB1-1/2/3 cell strains. This SNV will lead to a missense mutation, D736G. **F** The heterozygous SNV status of MA and MAB1 lymphomas arising from B10 recipient mice. AC1/2-specific SNVs were selected for clonal origin analysis of B10-MA/MAB1 lymphomas. Colored regions represent heterozygous single-nucleotide variants (SNVs), while blank areas indicate wild-type homozygous loci. Distinct colors denote cell line-specific heterozygous single-nucleotide variants (SNVs) in MA (orange) and MAB1 (blue) cells, which were utilized as molecular markers to reconstruct ancestral clone divergence patterns. **G** The diagram of the clonal origin of B10-MA and B10-MAB1 lymphomas.

In this study, we collected lymphoma samples from the four distinct groups of recipient mice mentioned earlier and isolated lymphoma-derived cell strains for transcriptome sequencing analysis (Fig. 3A). Notably, some of the samples were obtained from multiple anatomic locations in B6-MA-3 mice and B10-MAB1-1/2/3 mice. Subsequently, we conducted a comprehensive analysis of the transcriptome data, including the identification of SNVs, RNA editing events, and gene expression profiles (GEPs) (Fig. 3A).

Overall, the analysis of SNVs in both B6 and B10 mice revealed a higher number of SNVs in MAB1 lymphomas compared to MA lymphomas, as depicted in SNV patterns D and E (Fig. 3B). This suggests that EFNB1 plays a role in accelerating lymphoma clonal evolution. When comparing the two mouse strains, B10 lymphomas exhibited a greater number of SNVs than B6 lymphomas, as seen in SNV pattern C (Fig. 3B). This indicates that the genetic background of B10 mice has a more profound impact on lymphoma clonal evolution than that of B6 mice.

Notably, within the B10 background, the common SNVs between MA lymphomas and MAB1 lymphomas did not differ significantly, as shown in SNV pattern C. This suggests that the prolongation of tumor-bearing time does not significantly contribute to the accumulation of SNVs. Additionally, SNV pattern A represents the unique baseline SNVs that distinguish the MA/MAB1 cell lines from other samples, while SNV pattern B corresponds to the SNV pattern of AC1 described below.

A notable exception is the 2L2 clone in the B10-MAB1-2 mouse (Fig. 3B), which lacks the characteristic SNVs observed in other 2L1/3/4 clones. We hypothesize that this clone may be a lymphoma clone introduced during the injection process that resided and remained dormant in the tail during the early stages. Given the lack of clinical significance associated with tail colonization, we will refrain from further exploration.

Furthermore, our observations revealed identical clonal patterns among multiple lymphomas developing within the same recipient mouse in the MAB1-B10 model (Fig. 3B). This suggests that the systemic lymphomas originate from a single clone, indicating a clonal origin for these malignancies. We hypothesize that during lymphoma development, a specific clone acquires a survival advantage and evolves into a lymphoma, subsequently escaping immune surveillance, reshaping the immune system, and enhancing surveillance against other clones. We define this clone, which possesses a survival advantage and evolves into a tumor, as the dominant clone.

We categorize cases where tumors arise from different clones at various dissemination sites within an individual as the multiple-clone dissemination model. Conversely, when tumors arise from the same clone, we refer to it as the single-clone dissemination model (Supplementary Fig. 3A). In the context of solid tumors, multiple metastatic tumors may stem from distinct dominating clones, and tumor heterogeneity is a critical factor leading to tumor recurrence and drug resistance. If systemic lymphomas originate from a single dominant clone, then non-genetic factors are likely to underlie lymphoma recurrence, refractoriness, and heterogeneity. This important finding will provide profound insights for the clinical diagnosis and treatment of systemic lymphoma.

Next, we conducted a rigorous analysis of the distinct patterns of SNVs present in the MA and MAB1 cell lines. Specifically, we identified cell line-specific SNVs that persisted in the tumor as the signature SNVs of the ancestor clone (AC). Notably, our analysis revealed 17 unique SNVs in the MA cell line, consistently maintained in the MA/MAB1 lymphomas, designated as signature SNVs of AC1. Furthermore, 8 unique SNVs in the MAB1 cell line were also conserved in the MA/MAB1 lymphomas, labeled as signature SNVs of AC2. Based on these unique SNV signatures, it became evident that lymphomas arising in B6 mice from both MA and MAB1 could be traced back to either AC1 or AC2. Moreover, the SNV profiles of the MAB1 cell line exhibited a close resemblance to those of its originating lymphoma (Fig. 3C, D).

To further validate our findings, we selected a specific SNV site within the MAB1 cell line that caused a missense mutation, D736G, on the Ttk gene. We then verified its variant frequency across various samples. Since the SNV profiles were derived from transcriptome sequencing data, we designed both genomic DNA (gDNA) primers and cDNA primers, located in introns and exons, respectively, to amplify the DNA fragments encompassing the SNV site from both gDNA and cDNA for Sanger sequencing.

Firstly, the results demonstrated that the mutation frequencies of this SNV in gDNA and cDNA were similar (Fig. 3E), confirming that this SNV originated from the gDNA. Secondly, this SNV was absent in MA samples but emerged in the MAB1 cell line with a frequency below 50% (Fig. 3E). Notably, this SNV was present in MAB1-1L/2L at approximately 50% but undetectable in MAB1-3L (Fig. 3E). These results strongly suggest that MAB1-1L/2L lymphomas originated from AC2, while MAB1-3L lymphoma originated from AC1.

Drawing from the principle of Ig gene clonality analysis, which discriminates lymphoma from lymphoid hyperplasia in clinical molecular diagnosis, we conducted an Ig profile analysis of the MAB1 cell strains for clonality assessment. Seven Ig genes displaying distinct expression patterns in the MAB1-1/2/3 cell strains were selected, and their gDNA was analyzed (Supplementary Fig. 3B, C). The outcomes revealed that the MAB1-1/2/3 cell strains exhibited unique Ig rearrangement patterns.

Furthermore, we identified distinct SNV patterns observed in MAB1 lymphomas/cell strains and MA lymphomas (Supplementary Fig. 3D, E). Next, we selected three SNV sites uniquely expressed in the MAB1-1/2/3 cell strains and analyzed their mutation frequencies. The results demonstrated that the mutation frequencies of these specific SNVs in MAB1-1L/2L/3L lymphomas were approximately 50% (Supplementary Fig. 3F–H), indicating a heterozygous pattern and monoclonal origin. Notably, these SNVs were absent (Supplementary Fig. 3F, G) or present at low frequencies (Supplementary Fig. 3H) in the parental MAB1 cell line. The findings strongly suggest that the MAB1 cell line is heterogeneous, encompassing multiple clones. The MAB1 lymphomas/cell strains likely originated from preexisting clones within the MAB1 cell line and may have acquired novel SNVs during their development.

Finally, we analyzed the clonal relationship of lymphomas arising in B10 mice and found that MA lymphomas could originate from either the AC1 or AC2 clone. However, interestingly, all 10 MAB1 lymphomas traced their origin exclusively to the AC2 clone (Fig. 3F, G), suggesting that the AC2 clone of MAB1 exhibits a stronger tumorigenic potential in B10 mice. In essence, the genetic milieu of B10 mice confers a preferential selection advantage to the AC2 clone of MAB1, making it a more dominant player in lymphoma development within this mouse strain.

In summary, our clonality analysis reveals that both EFNB1 and the intrinsic differences among mouse strains can significantly influence the evolution and selection of lymphoma clones.

### EFNB1 manipulates RNA base substitutions with host heterogeneity

**Fig. 4:**
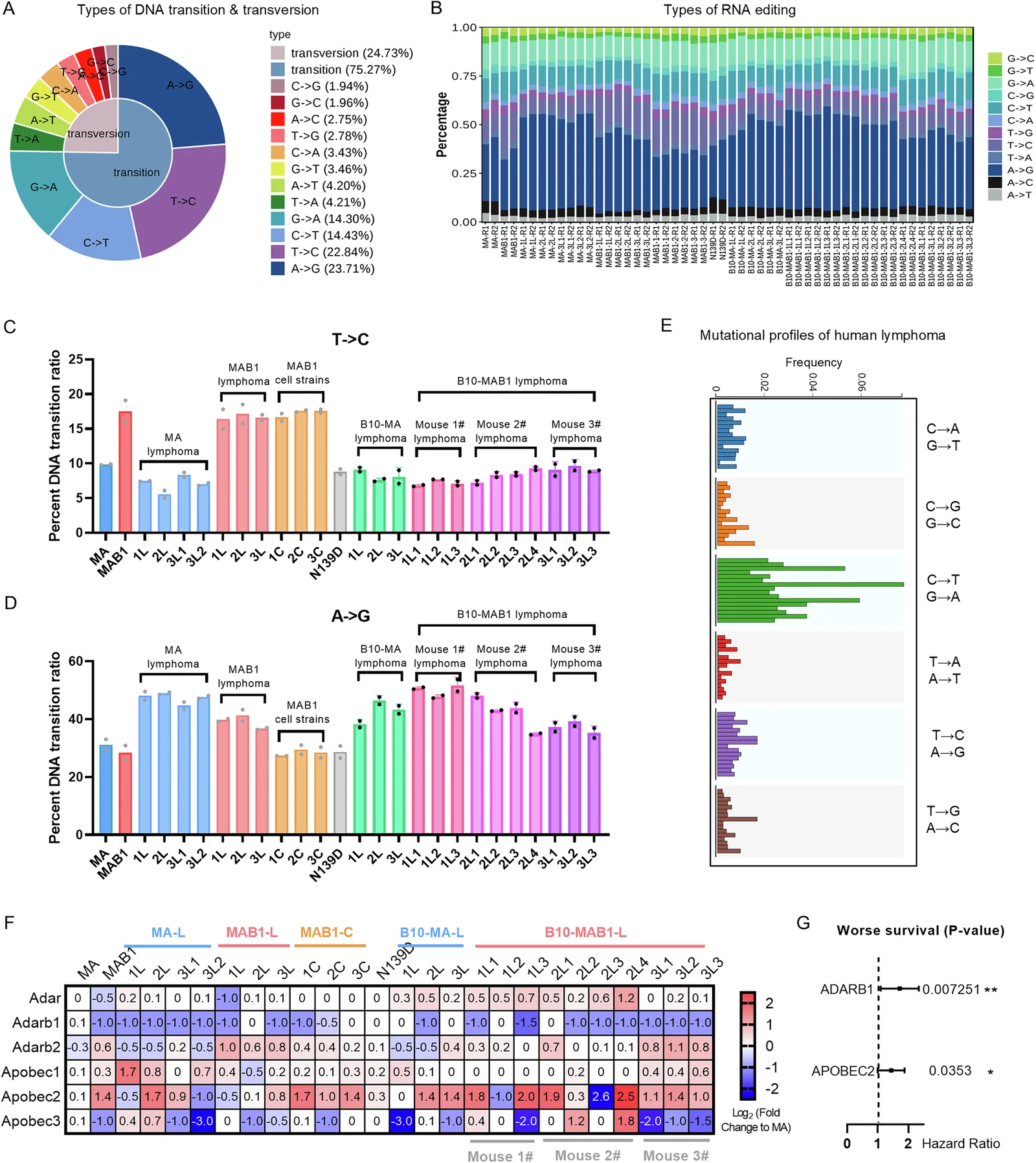
EFNB1 and host heterogeneity influence the ratio of RNA base substitutions. **A** The ratio of all types of DNA base substitutions within SNVs. The data from the MA cell line is presented, and the patterns observed in other samples are similar. **B** The ratio of all types of RNA base substitutions in all 26 samples. **C** The ratio of T->C substitution in all 26 samples. **D** The ratio of A->G substitution in all 26 samples. **E** Mutational signature of human lymphoma. Data was acquired from the MUTAGENE database. **F** The expression level of DNA/RNA deaminases in all 26 samples. The FPKM (fragment per kilobase of transcript per million mapped reads) value was used to quantify the expression abundance. All FPKM values were normalized to MA. **G** A forest plot illustrating the correlation between the expression levels of ADARB1 and APOBEC2 and their prognostic significance. Lenz Staudt DLBCL dataset (GSE10846, *n* = 414) was used for prognostic analysis.

To explore the influence of EFNB1 and mouse strains on sequence variations, we analyzed the frequencies of substitutions in SNVs and RNA editing events derived from transcriptome sequencing data. Our results demonstrated that there were no notable differences in the overall frequency of SNVs across the different groups (Fig. 4A). Nevertheless, noteworthy disparities emerged in the frequencies of RNA base substitutions across different groups of samples (Fig. 4B). Additionally, EFNB1-N139D, an inactive mutation that we recently identified, was employed in this study as a functional inactivation control.

The ratio of T-to-C substitutions was significantly elevated in the MAB1 cell line, B6-MAB1 lymphomas, and MAB1 cell strains compared to the MA cell line and B6-MA lymphomas, suggesting that overexpression of EFNB1 leads to an increase in T-to-C substitutions. However, interestingly, this increase in T-to-C substitutions triggered by EFNB1 was absent in B10-MAB1 lymphomas (Fig. 4C), suggesting that T-to-C substitutions mediated by EFNB1 are contingent on the inherent differences among host mouse strains.

The frequency of A-to-G substitutions was notably elevated in all lymphomas, but interestingly, it returned to levels comparable to those observed in the MA/MAB1 cell lines in the lymphoma-derived cell strains MAB1-1/2/3 (Fig. 4D). This finding suggests that the occurrence of A-to-G substitutions is regulated by the in vivo niche. Furthermore, the increase in A-to-G substitutions was more pronounced in B6-MA lymphomas compared to B6-MAB1 lymphomas (Fig. 4D), indicating that EFNB1 partially restricts the in vivo niche-mediated A-to-G substitution. However, in B10 lymphomas, the influence of EFNB1 was less pronounced (Fig. 4D). Additionally, the frequency of A-to-G substitutions in B10-MAB1 lymphomas exhibited variability among different mice, suggesting the existence of alternative mechanisms beyond EFNB1 and mouse strain differences. Moreover, mutational signatures revealed that C->T and G->A are the most prominent types of DNA base transitions in human lymphomas (Fig. 4E), highlighting the clinical relevance of changes in the ratio of RNA base substitutions observed in MA/MAB1 lymphomas.

RNA editing involves the insertion, deletion, or substitution of bases on RNA, and one of the mechanisms responsible for RNA base substitution is DNA or RNA deamination. A-to-G substitutions in mRNA are presumably caused by ADAR-mediated A-to-I editing, where ADARs convert adenosine to inosine, which is subsequently recognized as guanosine. Conversely, T-to-C substitutions in mRNA may arise from DNA mutations and other mechanisms, including ADAR-mediated RNA editing on antisense RNAs, which can lead to apparent T-to-C substitutions on overlapping sense transcripts [38]. APOBECs are crucial RNA/DNA deaminases that convert cytosine to thymine in DNA and uracil in RNA, resulting in C-to-T substitutions. Given the extraordinarily intricate nature of the mechanisms underlying RNA base substitutions [39, 40], which surpasses the scope of this study, we will refrain from further discussion.

To investigate the potential association between RNA deamination and RNA base substitutions, we analyzed the expression levels of ADARs and APOBECs across all lymphomas and cell lines/strains. Our findings revealed that, in comparison to the MA cell line, there were significant expression changes observed in endogenous ADARB1 and APOBEC2 in certain samples (Fig. 4F). However, these alterations did not exhibit a clear correlation with EFNB1 expression, mouse strains, ancestor clones, or the frequency of RNA base substitutions. Furthermore, prognostic analysis revealed a strong association between the expression levels of ADARB1 and APOBEC2 with poor prognosis in human DLBCL (Fig. 4G). A recent study has demonstrated that ADAR-mediated RNA editing can promote B-cell lymphomagenesis [41]. Given that ADARs possess the ability to modulate not only RNA editing but also transcript stability and gene expression [42], we hypothesize that the influence of ADAR levels on RNA base substitutions and lymphoma progression is likely to be highly intricate and multifaceted.

DNA mutations and RNA editing events are frequently observed to occur in a mutually exclusive manner in DLBCL [41], suggesting that RNA editing at the transcriptome level may serve as a complementary mechanism that drives tumor clonal evolution and malignant progression. RNA editing is believed to be an alternative mechanism underlying the transcriptome diversity in the pathogenesis of cancer [43–46]. Here, we demonstrated that EFNB1 can modulate the ratio of RNA base substitutions both in vitro and in vivo, exhibiting notable host heterogeneity. These findings suggest that RNA base substitutions function as a mechanism for EFNB1 to elicit transcriptome diversity, potentially explaining the diverse dissemination patterns observed in MAB1 lymphomas.

### EFNB1 contributes to the diversity of GEPs with host heterogeneity

Compared to genetic variations, GEPs serve as more intuitive markers for characterizing disease features. However, due to the poor RNA quality of clinically formalin-fixed paraffin-embedded samples, our understanding of GEPs and their association with lymphoma dissemination remains limited.

**Fig. 5:**
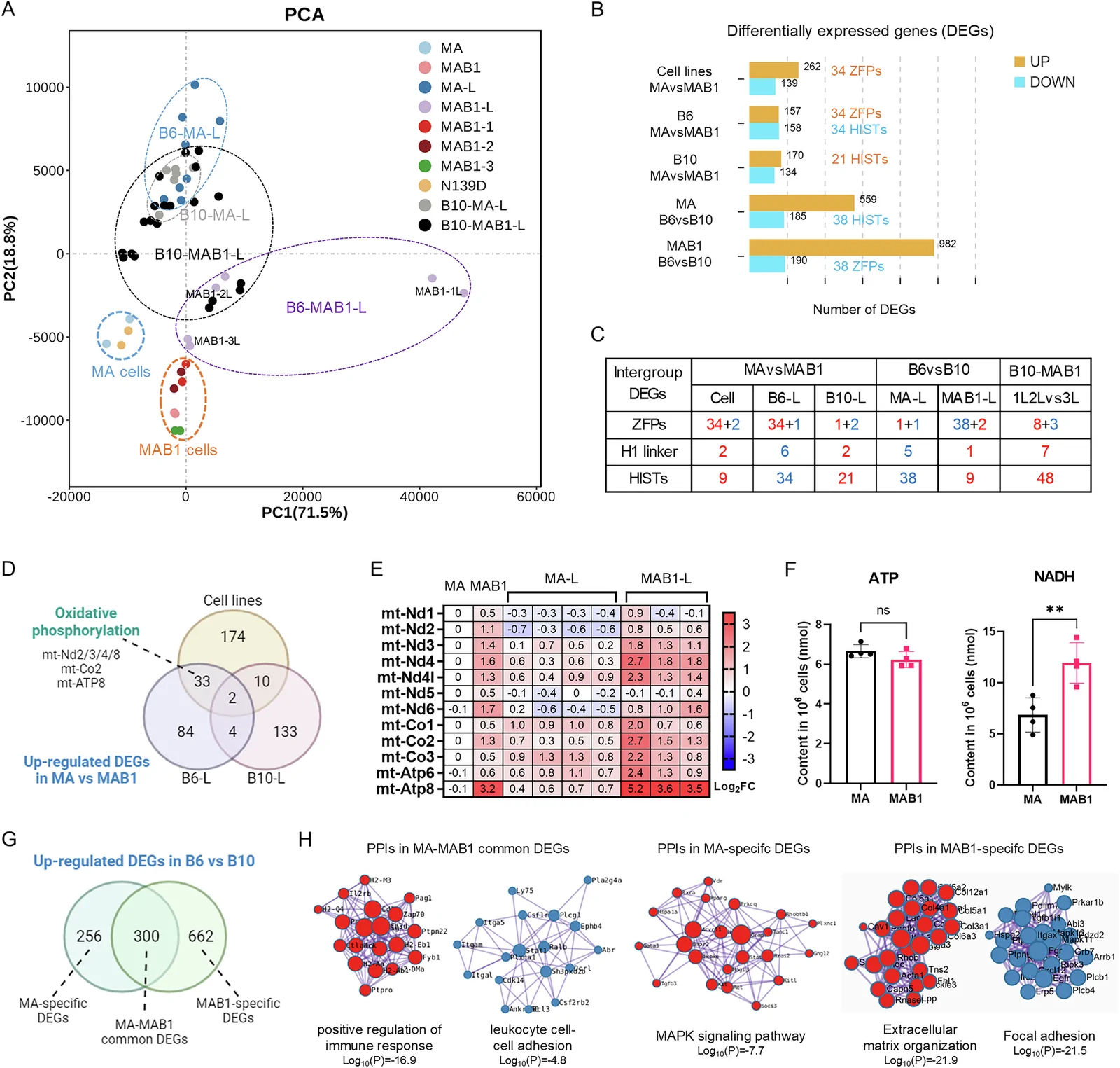
EFNB1 and host heterogeneity influence the transcription of nuclear histone genes and mitochondrial respiratory chain genes. **A** Principal component analysis (PCA) based on the expression profiles of all 26 samples. **B** The number of differentially expressed genes (DEGs) across five comparison groups. HISTs histone cluster genes, ZFPs Zinc finger proteins. **C** The number of ZFPs, H1 linker, and HISTs in each comparison group. Red, upregulated genes. Blue, down-regulated genes. **D** Upregulated DEGs in three comparison groups of MA versus MAB1. Multiple genes involved in oxidative phosphorylation were upregulated in MA versus MAB1 cell lines and B6 lymphomas. **E** The expression level of mitochondrial respiratory chain genes in MA/MAB1 cell lines and B6 lymphomas. All samples were normalized to the MA cell line. **F** ATP level and NADH content in MA and MAB1 cell lines. An unpaired *t*-test was used to analyze the statistical differences. Data are represented as mean ± SEM. **G** Upregulated DEGs in B6-L versus B10-L. **H** Enriched protein–protein interactions (PPIs) in MA-MAB1 common DEGs, MA-specific DEGs, and MAB1-specific DEGs.

To understand the impact of EFNB1 and mouse strains on the GEPs, we analyzed all MA/MAB1 lymphomas originating from B6 and B10 mice, as well as cell lines/strains. Sample relationship analysis revealed notable changes in the transcriptomes of MA/MAB1 lymphomas when compared to their respective cell lines/strains (Fig. 5A), suggesting transcriptional remodeling occurs during the process of lymphoma formation. Meanwhile, significant disparities were observed in the transcriptomes of B6-MA, B6-MAB1, B10-MA, and B10-MAB1 lymphomas (Fig. 5A), indicating that both EFNB1 and mouse strains contribute to the heterogeneity of lymphoma transcriptomes. Based on our sample relationship analysis, the transcriptomes of B6-MA and M10-MA lymphomas exhibit relatively minor differences, whereas significant variations were detected in the transcriptomes of B6-MAB1 and B10-MAB1 lymphomas (Fig. 5A), indicating that these variations may be attributed to the diversity of colonization sites of lymphomas.

Next, we analyzed the GEPs across five comparisons: MA versus MAB1 in cell lines, B6-lymphoma, and B10-lymphoma, as well as B6 versus B10 in MA lymphomas and MAB1 lymphomas. Notably, the number of DEGs in the two B6 versus B10 groups is obviously greater than that in the three MA versus MAB1 groups (Fig. 5B), suggesting the profound impact of mouse strains on GEPs.

During our analysis of these DEGs, we unexpectedly noticed a substantial upregulation or downregulation of a large number of histone genes (HISTs), H1 linker genes, and zinc finger proteins (ZFPs) across these comparisons (Fig. 5B, C, Supplementary Fig. 4A–C). Given the significant inter-mouse heterogeneity in the expression of histone genes specifically in B10-MAB1 lymphomas (Supplementary Fig. 4D), we further conducted a comparative analysis between B10-MAB1-1L/2L and B10-MAB1-3L lymphomas (Fig. 5C).

To validate the clinical relevance of the association between EFNB1 and histone genes, we analyzed the expression levels of histone H1 genes and histone H3 genes using a clinical gene expression dataset based on next-generation sequencing. The results revealed that the expression levels of 4 out of 7 histone H1 genes were associated with EFNB1 expression, with three of them exhibiting a positive correlation (Supplementary Fig. 5A). Additionally, the expression levels of 4 out of 8 histone H3 genes were associated with EFNB1 expression, all displaying a negative correlation (Supplementary Fig. 5B). Although the biological significance of these findings remains unclear, they indicate a strong clinical relevance between histone genes and the EFNB1 gene.

Subsequently, we conducted enrichment analysis on DEGs among the five comparisons. To exclude the interference of HISTs and ZFPs, we manually removed them from enrichment analysis. The results revealed that leukocyte-related pathways were enriched in DEGs of the three comparisons of MA versus MAB1 (Supplementary Fig. 6A–C). By intersecting the upregulated genes from the three groups, we observed that mitochondrial genes involved in oxidative phosphorylation were present in both cell line and B6-lymphoma comparisons (Fig. 5D). We further reviewed the expression levels of all mitochondrial oxidative phosphorylation genes and found that most of these genes were highly overexpressed in the MAB1 cell line and B6-MAB1 lymphomas (Fig. 5E). However, the increase in the expression levels of mitochondrial respiratory chain genes caused by EFNB1 did not occur in B10 mice (Supplementary Fig. 7A). Further analysis demonstrated that MA and MAB1 cell lines exhibited similar ATP levels but significant differences in NADH content (Fig. 5F), suggesting that EFNB1 may cause metabolic disorders in the oxidative respiratory chain.

To validate the clinical relevance of the association between EFNB1 and mitochondrial respiratory chain genes. We analyzed the expression levels of mt-ND genes and mt-ATP genes in the next-generation sequencing-based clinical gene expression dataset. The results showed that the expression levels of 8 out of 9 genes were significantly associated with EFNB1 expression (*p* < 0.0001, ****), displaying a negative correlation (Supplementary Fig. 7B). These findings strongly indicate that the association between mitochondrial respiratory chain genes and the EFNB1 gene holds significant clinical relevance.

In the two comparisons of B6 versus B10, we observed that the DEGs were primarily upregulated, indicating that the DEGs were mainly specific to the B10 in vivo niche. These upregulated genes were predominantly enriched in immune activation-related pathways such as leukocyte activation and immune response (Supplementary Fig. 6D, E). Furthermore, we divided the DEGs into 3 categories: MA-specific DEGs, MAB1-specific DEGs, and MA and MAB1 common DEGs. Subsequent pathway enrichment analysis and PPIs enrichment analysis revealed that the B10 in vivo niche exerted a distinctive influence on the GEPs of MA and MAB1 lymphomas (Fig. 5G, H, Supplementary Fig. 6F–I). These findings suggested that mouse strains, as the host intrinsic differences, can contribute to the heterogeneity observed in the transcriptional reprogramming of lymphomas.

Additionally, unlike solid tumors, lymphomas originate from monoclonal cells. The uncertainty caused by vector integration introduces a strong bias in clonal establishment, resulting in phenotypic heterogeneity. Previous studies have specifically discussed the integration copy number of *Eμ-Myc* in the original *Eμ-Myc* transgenic mouse model. In this study, we designed inverse PCR to analyze the integration sites of EFNB1 in cell clones and successfully identified integration sites in MAB1-1, MAB1-2, and MAB1-3. Results showed that no integration occurred in coding regions among all detected sites (Supplementary Fig. 8).

In summary, our findings reveal that both intrinsic factors of the tumor, such as clonal origin and the expression patterns of specific genes, as well as extrinsic factors like individual genetic differences and inter-individual variations, can reshape the GEPs of lymphomas. These diverse factors significantly contribute to the intricate diversity and heterogeneity observed in tumor transcriptomes.

### EFNB1 promotes B-lymphoma cell differentiation in vitro but not in vivo

The COO classification, a critical pathological determinant, serves as a key prognostic indicator for B-cell malignancies. To elucidate the role of EFNB1 in modulating lymphoma origin, we employed an RNA-seq-based algorithm [47, 48] to compute COO scores for *Eμ-Myc*-derived lymphomas and corresponding cell lines/strains. The COO score is derived from the expression levels of 20 genes associated with ABC/GCB subtypes, enabling precise discrimination between ABC- and GCB-derived lymphomas. Although directly applying a COO classification algorithm developed for human lymphoma sequencing data to murine lymphomas may not yield accurate COO subtype assignments, it remains feasible for comparative assessments of B-lymphoma cell differentiation states.

**Fig. 6:**
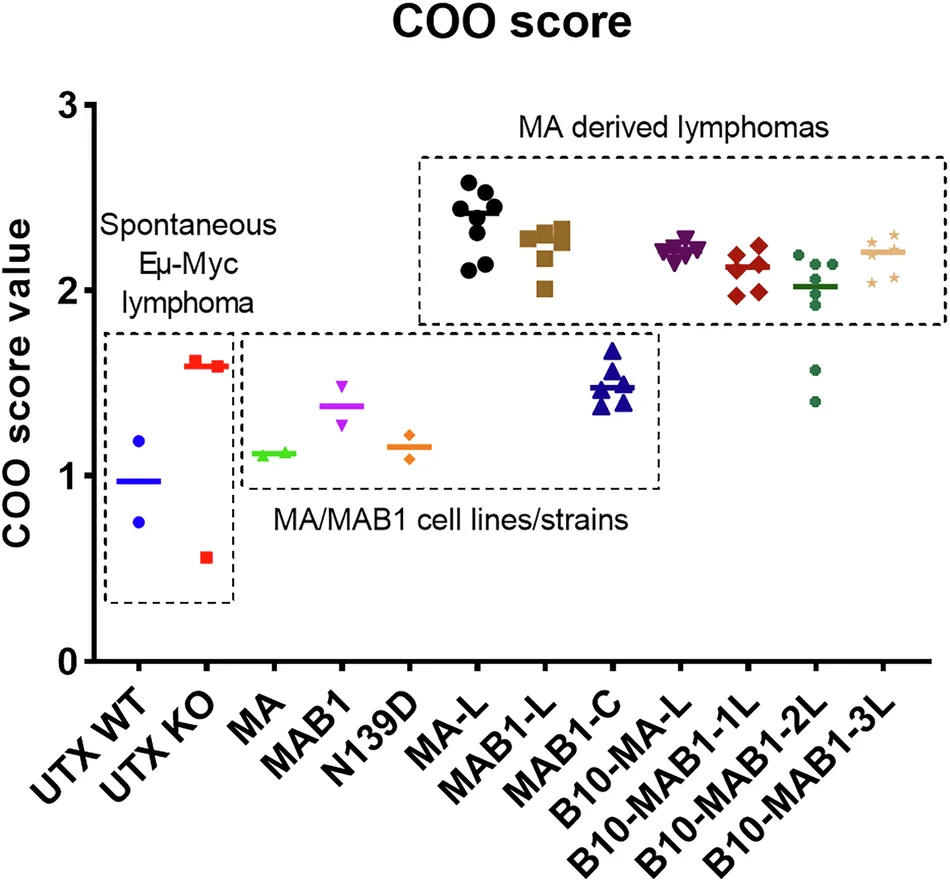
The Cell-of-Origin (COO) score of *Eμ-Myc*-derived lymphomas and corresponding cell lines/strains. Specifically, values for MA, MAB1, and N139D were calculated from technical replicates (*n* = 2), values for UTX WT, UTX KO, MA-L, MAB1-L/C, and B10-MA-L were computed using lymphoma samples from distinct mice, values for B10-MAB1-1L/2L/3L were determined from independent lymphomas isolated from the same mouse.

In this study, we computed the COO scores for original B-lymphoma cell lines, individual lymphomas, and their derived cell strains. The previously reported *Eμ-Myc* and *Eμ-Myc;UTX KO* lymphomas [14] were included as comparative controls in this study. The COO classification revealed that these samples predominantly exhibited an ABC-like phenotype (Fig. 6). Notably, *Eμ-Myc* and *Eμ-Myc;UTX KO* lymphomas displayed significantly lower COO scores compared to MA/MAB1-derived lymphomas, suggesting that MA lymphomas possess a higher degree of differentiation than the original *Eμ-Myc* lymphomas. Furthermore, MAB1 lymphoma-derived cell strains (MAB1-C) exhibited lower COO scores than their parental MAB1 lymphomas, implying that the in vivo niche is critical for maintaining the differentiation state of lymphomas. Additionally, both MAB1 cell lines and MAB1-C strains demonstrated higher COO scores than MA and N139D cell lines, indicating that EFNB1 overexpression promotes B-lymphoma cell differentiation in vitro.

### Identical clonal origin does not determine the same organotropism

At the cellular level, the stability of the genome is relative, while mutation and evolution occur continuously. Similarly, the transcriptome of a cell population should be in a relatively steady state and undergo transformation in response to specific signals, known as tumor cellular plasticity. Therefore, although the MAB1-1/2/3 cell lines originate from monoclonal MAB1 lymphoma, the cells they consist of are not completely identical and are in a state of dynamic change.

**Fig. 7:**
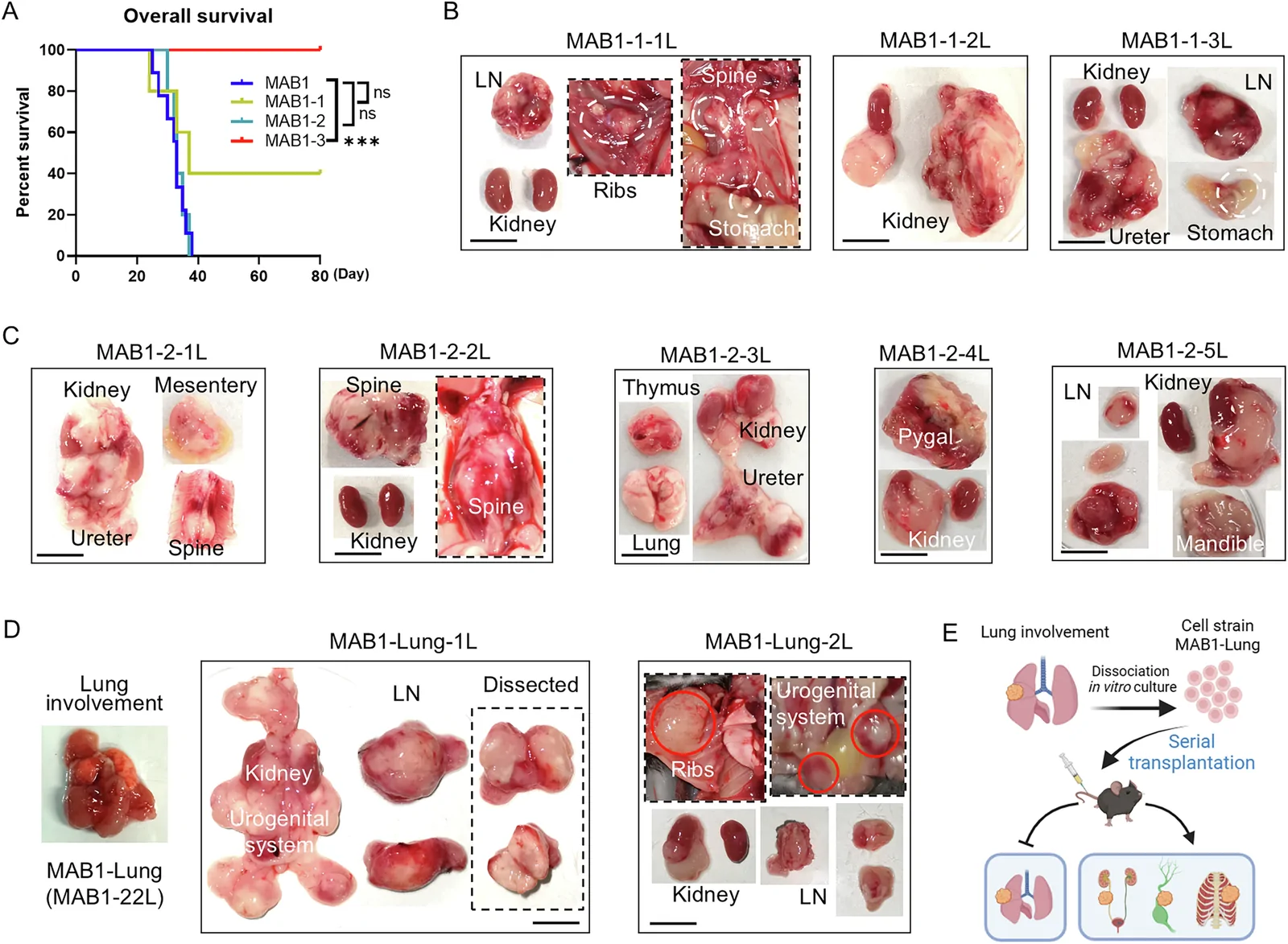
Monoclonal MAB1 cell strains originating from MAB1 lymphoma lack specific organ tropism in the serial transplantation model. **A** Survival curve of the B6 recipient mice transplanted with the parental MAB1 cell line and MAB1-1/2/3 cell strains. *n*(MAB1) = 9, *n*(MAB1-1/2/3) = 5. The log-rank (Mantel–Cox) test was used to evaluate the survival curves’ significance. **B** Representative images of extranodal colonization of MAB1-1 lymphoma. **C** Representative images of extranodal colonization of MAB1-2 lymphoma. **D** Representative images of extranodal colonization of MAB1-Lung lymphoma. MAB1-Lung cell strain is derived from MAB1-22L lymphoma. **E** Organ tropism of the MAB1-Lung cell strain in the serial transplantation model. Bar, 10 mm. The image within the dashed outline is not presented to scale.

To investigate whether the lymphoma clone itself is a key determinant of organotropism, we analyzed organotropism of the MAB1-1/2/3 cell strains in serial transplantation experiments, which are commonly used to verify the transplantability of tumor cells and thereby assess their tumorigenicity. Surprisingly, the incidence of MAB1-1/2/3 lymphoma in B6 recipient mice was not the same, and the features of organotropism were also different. Only about half of the MAB1-1 transplanted mice developed lymphomas (Fig. 7A). The tumorigenicity and overall survival time of MAB1-2 were similar to those of the parental MAB1 cell line (Fig. 7A). MAB1-3 completely lost its tumorigenicity (Fig. 7A).

In terms of organ tropism, both MAB1-1 lymphoma and MAB1-2 lymphoma exhibit non-specific dissemination sites, exhibiting significant individual variability and randomness (Fig. 7B, C). We further analyzed the organotropism of the MAB1-lung cell strain, exhibiting prominent lung tropism. The results showed that MAB1-lung lymphoma involved multiple organs in two recipient mice, rather than the lungs (Fig. 7D, E). Additionally, we had isolated three monoclonal MA cell lines in vitro and verified their tumorigenicity by serial transplantation, 3 recipient mice for each MA clone. The results showed that most recipient mice (8/9) failed to develop lymphomas (Supplementary Table 1). In our experience, monoclonal cell strains derived from in vitro limited dilution often fail to tumorigenize. In contrast, cell strains established from most lymphomas through ex vivo culture typically retain tumorigenic potential upon serial transplantation, suggesting in vivo clonal selection and evolutionary reprogramming are critical determinants of lymphoma clonal tumorigenicity. However, notable exceptions have been observed: the MA-1 cell line (derived from the MA-1L lymphoma) cannot be propagated in vitro, and the MAB1-3 cell line (derived from the MAB1-3L lymphoma) fails to form tumors in vivo.

In summary, these results demonstrate that an identical clonal origin, as defined by shared SNV signatures, is neither sufficient nor predictive for a specific organotropism pattern, highlighting the critical role of post-transplantation evolutionary dynamics and microenvironmental selection. Persistent clonal evolution and transcriptome reshaping are reasons for the heterogeneity and the diversity of lymphoma organotropism. Due to the absence of selection pressure for tumorigenicity, the in vitro reshaping may lead to the emergence of clones that lose their tumorigenicity. Therefore, we propose that high homogeneity of cell populations will reduce the incidence of tumorigenesis, and maintaining heterogeneity of cell populations is essential for tumor incidence. The higher the heterogeneity of tumor cells, the higher the probability that specific clones will survive and form tumors after experiencing selection and adaptation in the in vivo niche. It is noteworthy that, unlike the metastasis process of primary tumors, transplantation of homogenous tumor cells is a highly artificial experiment, which does not represent the true pathological process of tumorigenesis.

## Discussion

**Fig. 8:**
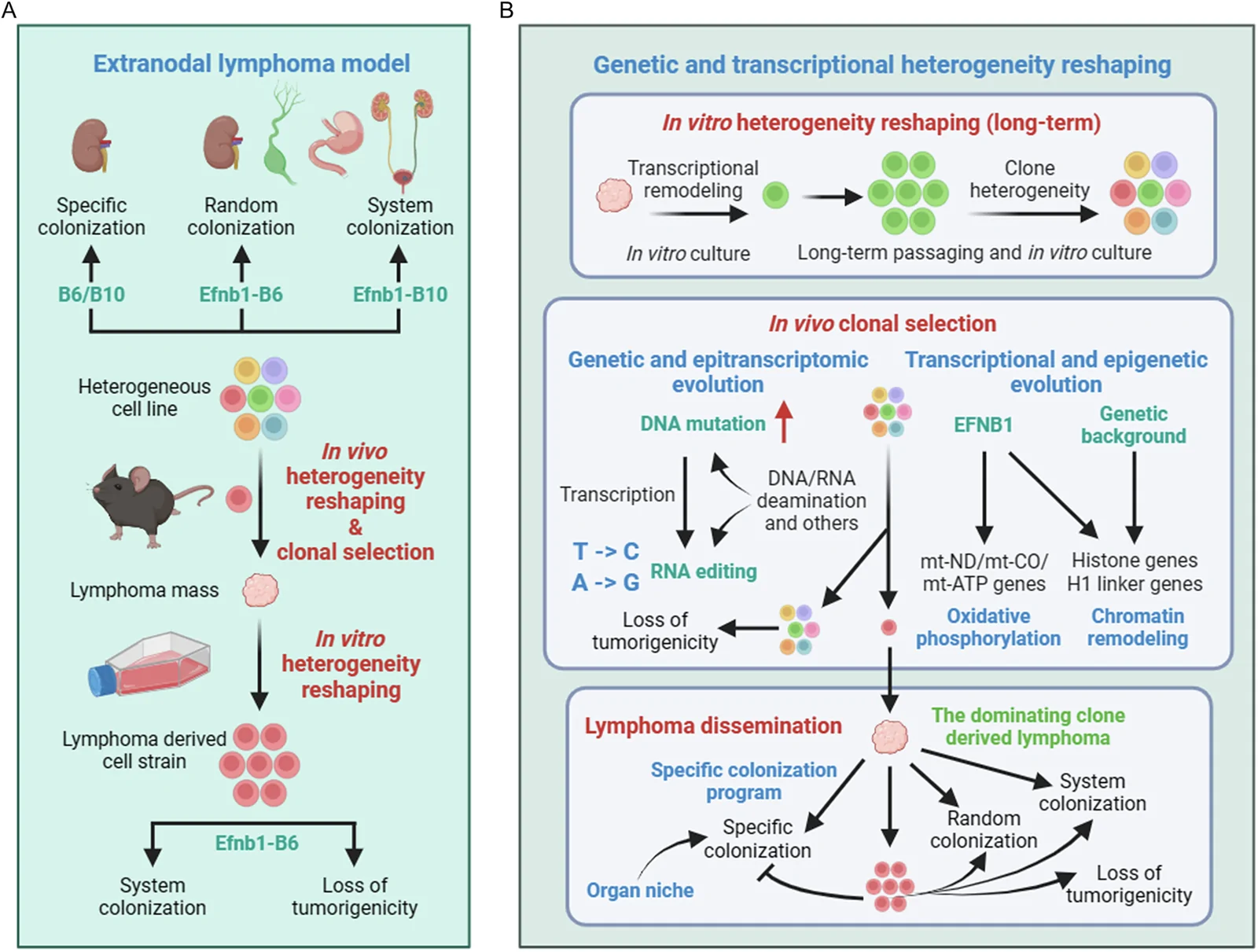
The extranodal lymphoma model elucidates the reshaping of genetic and transcriptional heterogeneity within the tumor during lymphoma progression and dissemination. **A** Schematic illustrations depicting the influence of EFNB1 and mouse strains on lymphoma dissemination within the extranodal lymphoma model in this study. **B** The reshaping of genetic and transcriptional heterogeneity during in vitro culture and in vivo selection influences lymphoma dissemination.

In this study, we successfully overexpressed EFNB1 in MA cell lines, leading to the establishment of the MAB1 cell model, which altered the lymphoma’s tropism, shifting from a specific organ tropism to a random multiple organ tropism or systemic dissemination. This finding underscores the pivotal role of EFNB1 in the malignant progression of lymphoma. We described the disparities and interconnections among lymphoma clones at both genomic and transcriptional levels during lymphoma dissemination across two different mouse strains (Fig. 8A, B). Consistent with our recent proposal, lymphoma dissemination is a hallmark of its malignant progression [49]. Compared with nodal lymphoma, extranodal lymphoma is likely caused by the loss of the ability to colonize lymph nodes. Given current knowledge of epigenetic regulation and mitochondrial function in cancer [50, 51], we propose that EFNB1 signaling may regulate histone gene expression, thereby shaping the epigenetic landscape and modulating mitochondrial gene expression and metabolic reprogramming. This cascade ultimately impairs the ability of lymphoma cells to colonize lymph nodes and promotes their dissemination to multiple organs. Moreover, this process appears to be context-dependent and may vary among individuals with different genetic backgrounds.

In this study, the utilization of B6 and B10 strains for evaluating host heterogeneity is rather restrictive. Both B6 and B10 are inbred strains that have undergone artificial cultivation, leading to considerable divergence from their wild counterparts. While C57 inbred strains indeed possess experimental advantages in terms of tumorigenicity, the significant absence of genetic diversity significantly undermines the reliability of the conclusions drawn from them [52]. It is crucial to acknowledge that cancer is an exceedingly intricate disease, arising from a confluence of multiple factors [53]. The development of advanced tumor models serves as a pivotal foundation for comprehensively grasping the intricate pathological processes involved. The isolation and identification of a greater number of transplantable murine cell lines, leading to the establishment of diverse CellDAs models with varying genetic backgrounds, will facilitate a more comprehensive analysis of tumorigenesis and tumor clonal evolution.

Canonical histone transcripts were previously believed to lack poly(A) tails [54], leading to the assumption that they should not be detectable in poly(A)-enriched RNA sequencing. However, contrary to this belief, numerous GEPs datasets obtained through next-generation sequencing, which relies on poly(A) enrichment, have revealed that over half of the histone genes are detectable with high levels of reads. This finding suggests that most histone genes actually possess poly(A) tails, as also confirmed by a recent study [55]. Given that histone genes are clustered together [56], we hypothesize that they may be arranged tandemly and transcribed jointly, sharing a common poly(A) tail, similar to the polycistron in prokaryotes. This hypothesis enables multiple histone genes to respond to specific signals, such as the EFNB1 signal, and be transcribed simultaneously. Numerous studies have reported that elevated expression levels of histone genes are associated with poor prognosis in tumor patients [57–60]. Moreover, mutations in the histone H1 have been implicated in lymphomagenesis, likely through disruption of chromatin organization [61, 62]. Given their critical roles in nucleosome assembly and chromatin remodeling, we postulate that the regulation of histone contents serves as a mechanism that influences tumor progression. In this study, both EFNB1 and host heterogeneity contribute to the transcriptional regulation of histone genes. Furthermore, this regulation may represent one of the mechanisms by which EFNB1 and host heterogeneity modulate transcriptome diversity at the epigenetic level.

A recent study has revealed that mitochondrial DNA mutations can regulate tumor metabolism and checkpoint blockade response by promoting aerobic glycolysis [63], suggesting that the transcriptional regulation of mitochondrial respiratory chain genes governed by EFNB1, as discovered in our study, may hold a pivotal biological role in lymphoma progression. Furthermore, another study has demonstrated that the increased histone density in cancer cells affects the composition of the mitochondrial proteome [64], hinting at the existence of an intricate regulatory network encompassing EFNB1, histone genes, and mitochondrial genes.

We believe that host heterogeneity manifests across multiple dimensions, including immunity and metabolism. Taking the C57BL/6 and C57BL/10 mouse strains as examples, they exhibit well-documented differences in immunity and metabolism. For example, B10 mice are deficient in complement C5 [65], whereas B6 mice carry a mutation in the Nnt gene associated with glucose metabolism [66]. Identifying which specific aspect influences lymphoma dissemination will be a highly challenging endeavor. Differences in immune function may indeed represent an important aspect of host heterogeneity. However, immune competence is difficult to assess in human populations. Although age-related differences in immune function are evident, variations among individuals of similar age remain highly challenging to evaluate. We propose that metabolic differences may represent a key aspect of host heterogeneity that is both clinically relevant and experimentally tractable. For instance, obesity status is relatively easy to assess in human populations and can be linked to metabolic parameters and associated diseases. Experimentally, high-fat diet feeding is also highly feasible. Taken together, we suggest that future investigations into host heterogeneity could reasonably focus on metabolic differences—for example, by using aggressive lymphoma models or malignant solid tumor models to explore clinically relevant host factors such as metabolic status or obesity, and their impact on lymphoma dissemination, distant metastasis, and organotropism.

This study explored the influence of host heterogeneity on lymphoma progression and its transcriptional implications within the framework of the established MAB1 model. Although the lymphoma characteristics, particularly the organ tropism, closely mirrored those observed in clinical settings, this significantly escalated the complexity and intricacies involved in experimental design and data analysis. Especially, the scarcity of detailed extranodal dissemination information in most existing B-cell lymphoma gene expression datasets hampers our efforts to establish clinical correlations for the findings of this study. The limited number of samples analyzed in this study was also inadequate to comprehensively capture the nuances of the model. Meanwhile, both strains of recipient mice used in this study, despite their suitability for establishing the MAB1 lymphoma model, are inbred lines maintained under controlled experimental conditions and do not reflect the natural population.

Current preclinical tumor models used for drug efficacy evaluation still primarily rely on tumor growth inhibition as the key criterion, limiting the discovery of antitumor drugs that can prevent tumor metastasis [67]. The MAB1 PEL model established in this study, exhibiting multi-organotropism, serves as an exemplary model for aggressive B-cell lymphoma. Meanwhile, the PEL model represents a promising tool for exploring tumor heterogeneity and clonal evolution. Additionally, the MAB1 PEL model also serves as a preclinical “clinical trial” model for evaluating the effectiveness of anticancer therapies, including immunotherapy. The utilization of the MAB1 PEL model is anticipated to refine evaluation criteria and boost prediction accuracy for potential antitumor drugs, ultimately contributing to a higher success rate in clinical trials.

## Supplementary information

Supplementary Information

## Data Availability

All raw data generated or analyzed throughout this study are accessible upon reasonable request from the corresponding author. RNA-Seq data pertaining to this investigation have been deposited in the NCBI Sequence Read Archive (SRA) under the following accession codes: PRJNA780031 (B6-MA), PRJNA763338 (B6-MAB1), PRJNA1102933 (B10-MA), and PRJNA1104347 (B10-MAB1). Requests for materials should be directed to the lead contact. Any cell lines or strains generated in this study can be acquired through the Materials Transfer Agreement (MTA). Further information and requests for resources and reagents should be directed to XL (lixiaoxi@ujs.edu.cn).
