# Supplementary material for: Host heterogeneity shapes clonal selection and organotropism in an extranodal lymphoma mouse model
Source: Cell Death Dis. 2026 Jul 10;17(1):856. doi: 10.1038/s41419-026-09014-3 (PMC13635245; doi:10.1038/s41419-026-09014-3)
Supplement: Supplementary file 1 [file 41419_2026_9014_MOESM1_ESM.pdf]

Supplementary Information

Host heterogeneity shapes clonal selection and organotropism in an extranodal lymphoma mouse model

Xiaoxi Li *et al.*

Corresponding authors: lixiaoxi@ujs.edu.cn (Xiaoxi Li)

List of Supplementary Materials

Supplementary Figure 1–8
Supplementary Table 1-2

Figure S1. Lymphoma kidney-tropism in MA and MAB1 models.
Figure S2. Histological analysis of MA and MAB1 lymphomas.
Figure S3. The clone dissemination models and the validation of the clonal origin of B6-MAB1 lymphoma.
Figure S4. The expression patterns of histone cluster genes across various samples.
Figure S5. The expression levels of EFNB1 correlate with those of histone H1 and histone H3.
Figure S6. Enriched pathways of DEGs in each comparison group.
Figure S7. The expression levels of EFNB1 correlate with those of mitochondrial respiratory chain genes.
Figure S8. Retroviral integration site analysis by inverse PCR.

Table S1. Information of syngeneic lymphoma reconstruction experiments.
Table S2. Primers for SNVs verification and Ig rearrangement analysis.

A

Lymphoma kidney-tropism in MA-EV model

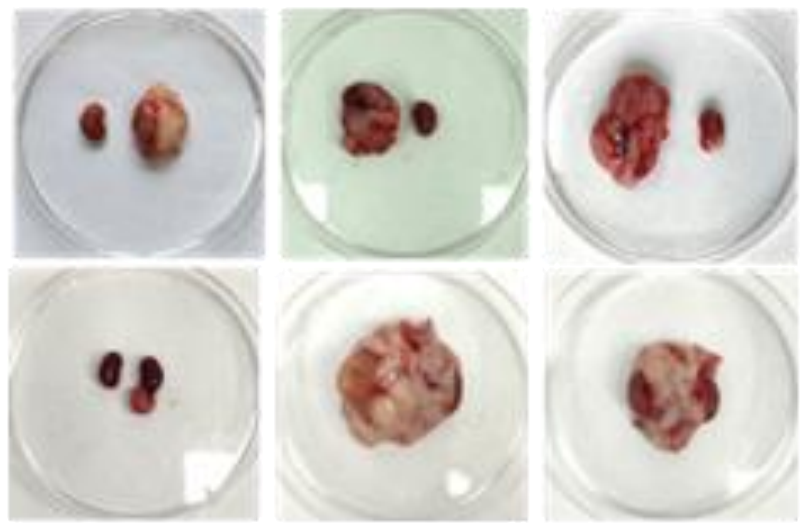

B

Lymphoma kidney-tropism in MA and MA-EV

Extranodal lymphoma	MA	MA-EV	Total
Kidney-only	4	6	10
Non-Kidney	0	0	0
Total	4	6	10

$p>0.999$ ns (Fisher's exact test)

C

Lymphoma kidney-tropism of B6-MA and B6-MAB1

Extranodal lymphoma	MA	MAB1	Total
Kidney-only	4	0	4
Non-Kidney	0	4	4
Total	4	4	8

$p=0.0286$ * (Fisher's exact test)

D

Lymphoma kidney-tropism in B6-MA and B10-MA

Extranodal lymphoma	B6-MA	B10-MA	Total
Kidney-only	4	3	7
Non-Kidney	0	1	1
Total	4	4	8

$p>0.999$ ns (Fisher's exact test)

Figure S1. Lymphoma kidney-tropism in MA and MAB1 models. A. Pictures of extranodal dissemination of lymphoma in MA-EV lymphoma mice. B-D. Incidence of kidney involvement by lymphoma in different models.

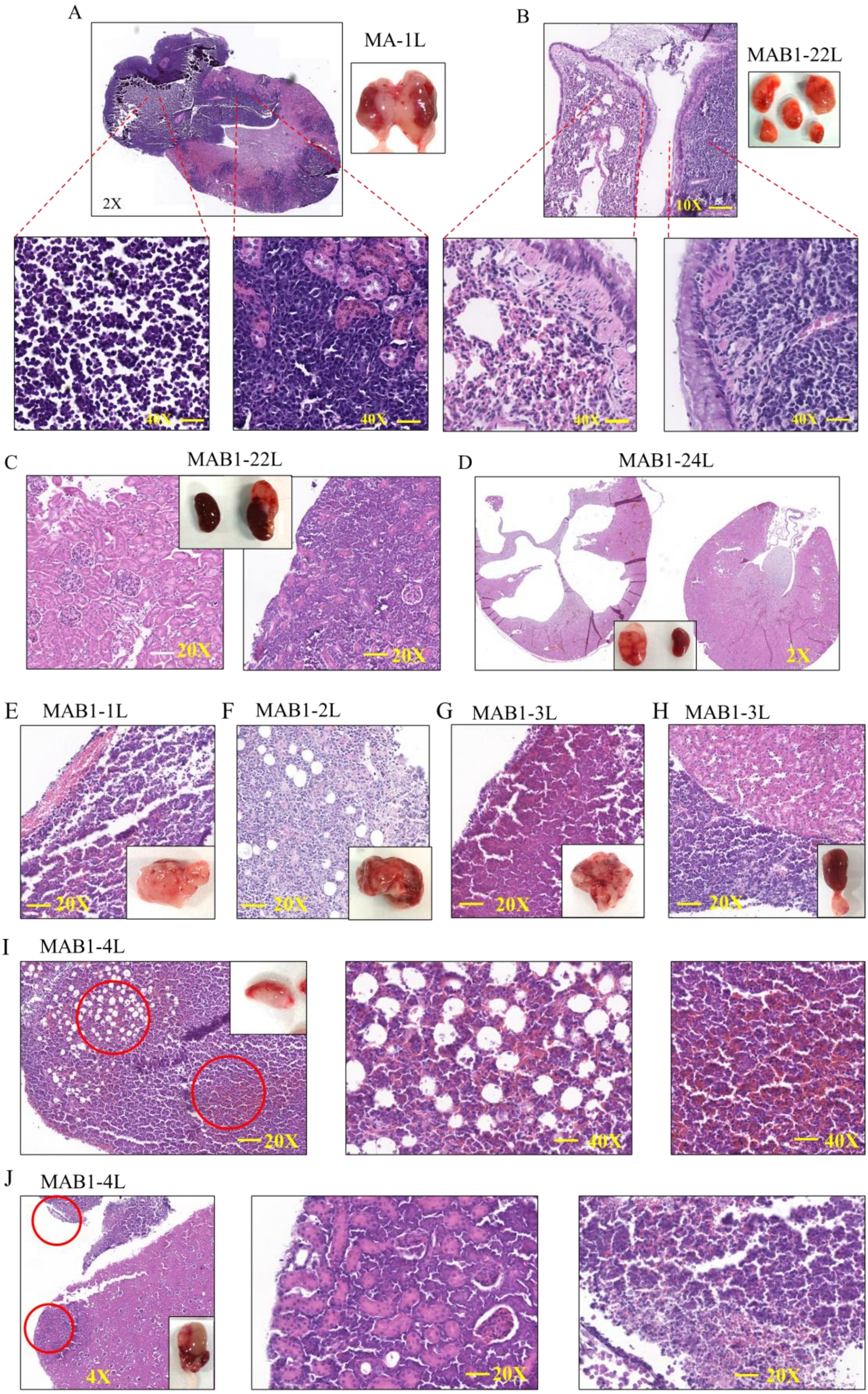

Fig. S2. Histological analysis of MA and MAB1 lymphomas. At a magnification of 20×, the bar scale corresponds to 200 nm; at 40×, it corresponds to 400 nm.

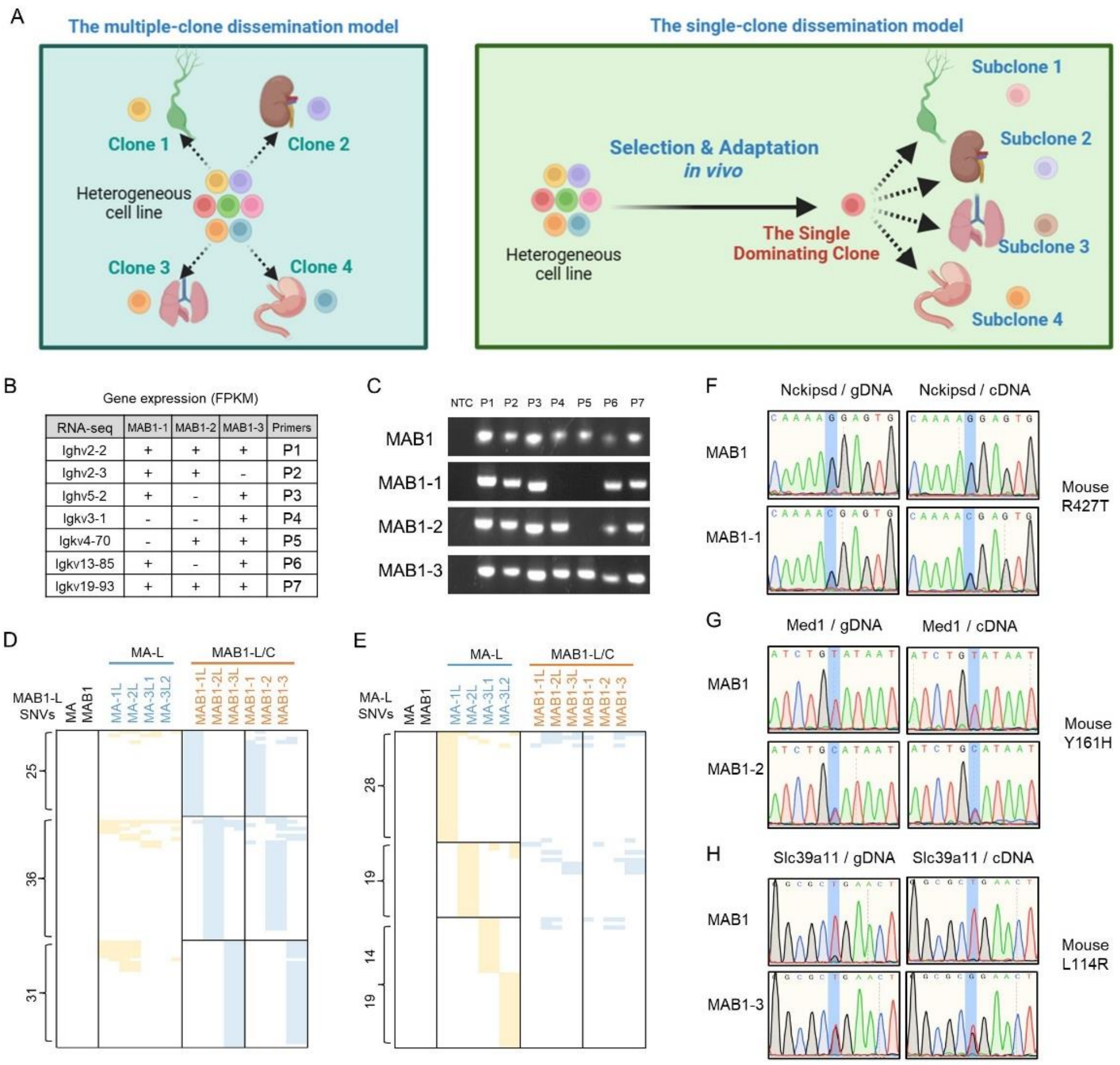

Fig. S3. The clone dissemination models and the validation of the clonal origin of B6-MAB1 lymphoma. A. Diagrams of the multiple-clone dissemination model and the single-clone dissemination model. These two models are employed to illustrate the clonal origin relationships among lymphomas that disseminate to multiple anatomical locations within a single individual. B. The expression status of 7 Immunoglobin (Ig) genes in MAB1-1/2/3 cell strains. '+', high expression. '-', low/none expression. C. PCR analysis of the gDNA rearrangement patterns of Ig genes in parental MAB1 cell line and MAB1-1/2/3 cell strains. The forward and reverse primers of Ig genes were designed on intron 1-2 and exon 2, respectively. D. The MAB1-L specific SNVs. SNVs that are absent in MA/MAB1 but newly emerge in MAB1 lymphomas are considered specific SNVs for MAB1 lymphomas. MAB1-L, MAB1 lymphoma. Colored regions represent heterozygous single nucleotide variants (SNVs), while blank areas indicate wild-type homozygous loci. Colors denote distinct categories of lymphoma subtypes or cell lines. E. The MA-L specific SNVs. SNVs that are absent in MA/MAB1 but newly emerge in MA lymphomas are considered specific SNVs for MA lymphomas. MA-L, MA lymphoma. F-H. Sequencing validation of the frequency of three SNV loci in MAB1-1/2/3 cell strains, respectively. These SNVs will lead to missense mutations,

R427T on Nckipsd gene in MAB1-1 cell strain, Y161H on Med1 gene in MAB1-2 cell strain, L114R on Slc39a11 gene in MAB1-3 cell strain.

A

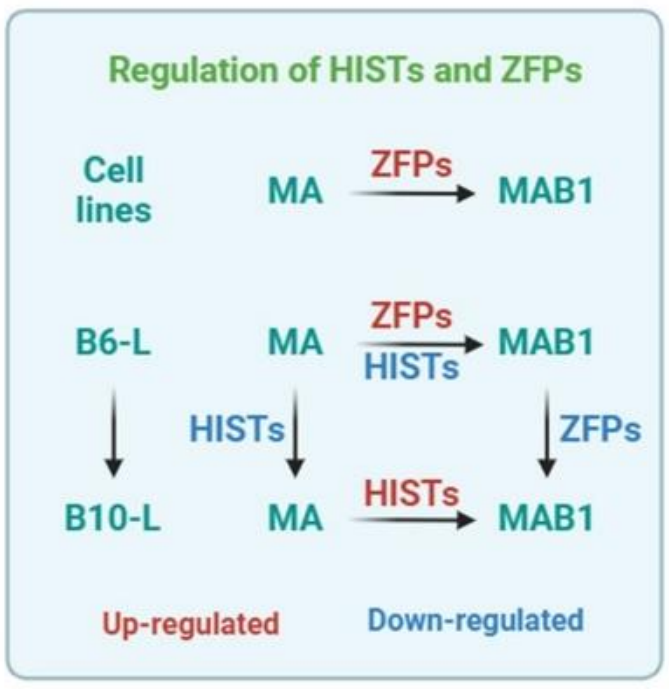

B

Intergroup DEGs	MAvsMAB1			B6vsB10		B10-MAB1
	Cell	B6-L	B10-L	MA-L	MAB1-L	1L2Lvs3L
H1 linker	2	6	2	5	1	7
HISTs	9	34	21	38	9	48
H2A	3	9	4	9	3	13
H2B	2	11	8	12	1	14
H3	1	8	6	9	4	11
H4	3	6	3	8	1	10

C

Intergroup DEGs	Cell	B6-L	B10-L	B10-L	MA-L	MAB1-L	Expression level
	MAvsMAB1			1L2Lvs3L	B6vsB10		
H2ac10	-	H2ac10	-	H2ac10	-	-	Medium
H2ac11	-	H2ac11	-	H2ac11	H2ac11	-	Medium
H2ac12	-	H2ac12	-	H2ac12	H2ac12	-	Medium
H2ac13	-	H2ac13	-	-	H2ac13	-	Medium
H2ac15	-	-	-	H2ac15	-	-	Low
H2ac18	H2ac18	H2ac18	-	H2ac18	H2ac18	H2ac18	High
H2ac19	-	-	-	H2ac19	-	-	Medium
H2ac22	-	-	-	H2ac22	-	-	Medium
H2ac24	-	H2ac24	H2ac24	H2ac24	H2ac24	H2ac24	Medium
H2ac4	-	-	-	H2ac4	H2ac4	-	Medium
H2ac6	H2ac6	H2ac6	H2ac6	H2ac6	H2ac6	-	Medium
H2ac7	-	H2ac7	H2ac7	H2ac7	H2ac7	-	Medium
H2ac8	-	H2ac8	H2ac8	H2ac8	H2ac8	H2ac8	Medium
H2aw	H2aw	-	-	H2aw	-	-	Medium
H2bc11	-	-	-	H2bc11	-	-	Low
H2bc12	-	-	-	H2bc12	H2bc12	-	Low
H2bc14	-	-	-	H2bc14	-	-	Low
H2bc15	-	H2bc15	-	H2bc15	H2bc15	H2bc15	Low
H2bc21	H2bc21	H2bc21	H2bc21	H2bc21	H2bc21	-	High
H2bc22	-	H2bc22	-	H2bc22	H2bc22	-	Medium
H2bc23	-	H2bc23	H2bc23	H2bc23	H2bc23	-	Medium
H2bc24	-	H2bc24	H2bc24	H2bc24	H2bc24	-	Medium
H2bc3	-	H2bc3	H2bc3	H2bc3	H2bc3	-	Medium
H2bc4	-	H2bc4	-	H2bc4	H2bc4	-	High
H2bc6	-	H2bc6	H2bc6	H2bc6	H2bc6	-	Medium
H2bc7	-	H2bc7	H2bc7	H2bc7	H2bc7	-	Medium
H2bc8	-	H2bc8	H2bc8	H2bc8	H2bc8	-	High
H2bc9	H2bc9	H2bc9	H2bc9	H2bc9	H2bc9	-	Medium
H3c1	-	H3c1	-	H3c1	H3c1	-	Medium
H3c10	-	-	-	H3c10	-	-	Low
H3c11	-	H3c11	H3c11	H3c11	H3c11	H3c11	Medium
H3c14	H3c14	H3c14	H3c14	H3c14	H3c14	-	Medium
H3c15	-	-	-	H3c15	-	-	Medium
H3c2	-	H3c2	H3c2	H3c2	H3c2	-	Medium
H3c3	-	H3c3	H3c3	H3c3	H3c3	H3c3	Medium
H3c4	-	H3c4	H3c4	H3c4	H3c4	-	Medium
H3c6	-	H3c6	H3c6	H3c6	H3c6	H3c6	Low
H3c7	-	-	-	H3c7	-	-	Low
H3c8	-	H3c8		H3c8	H3c8	H3c8	Medium
H4c1	-	H4c1	H4c1	H4c1	H4c1	-	Low
H4c11	-	H4c11	-	H4c11	H4c11	-	Low
H4c12	-	-	-	H4c12	-	-	Low
H4c14	-	-	-	H4c14	H4c14	-	Low
H4c18	H4c18	H4c18	-	H4c18	H4c18	-	Medium
H4c3	-	H4c3	H4c3	H4c3	H4c3	H4c3	Low
H4c4	H4c4	H4c4	H4c4	H4c4	H4c4	-	Medium
H4c8	-	H4c8	-	H4c8	H4c8	-	Medium
H4c9	-	-	-	H4c9	H4c9	-	High
H4f16	H4f16	-	-	H4f16	H4f16	-	Low
H1f0	-	H1f0	-	H1f0	-	-	High
H1f1	-	H1f1	-	H1f1	-	H1f1	Medium
H1f2	-	H1f2	-	H1f2	H1f2	-	High
H1f3	H1f3	H1f3	H1f3	H1f3	H1f3	-	Medium
H1f4	H1f4	H1f4	H1f4	H1f4	H1f4	-	Medium
H1f5	-	H1f5	-	H1f5	H1f5	-	Medium
H1f6	-	-	-	H1f6	H1f6	-	Low

D

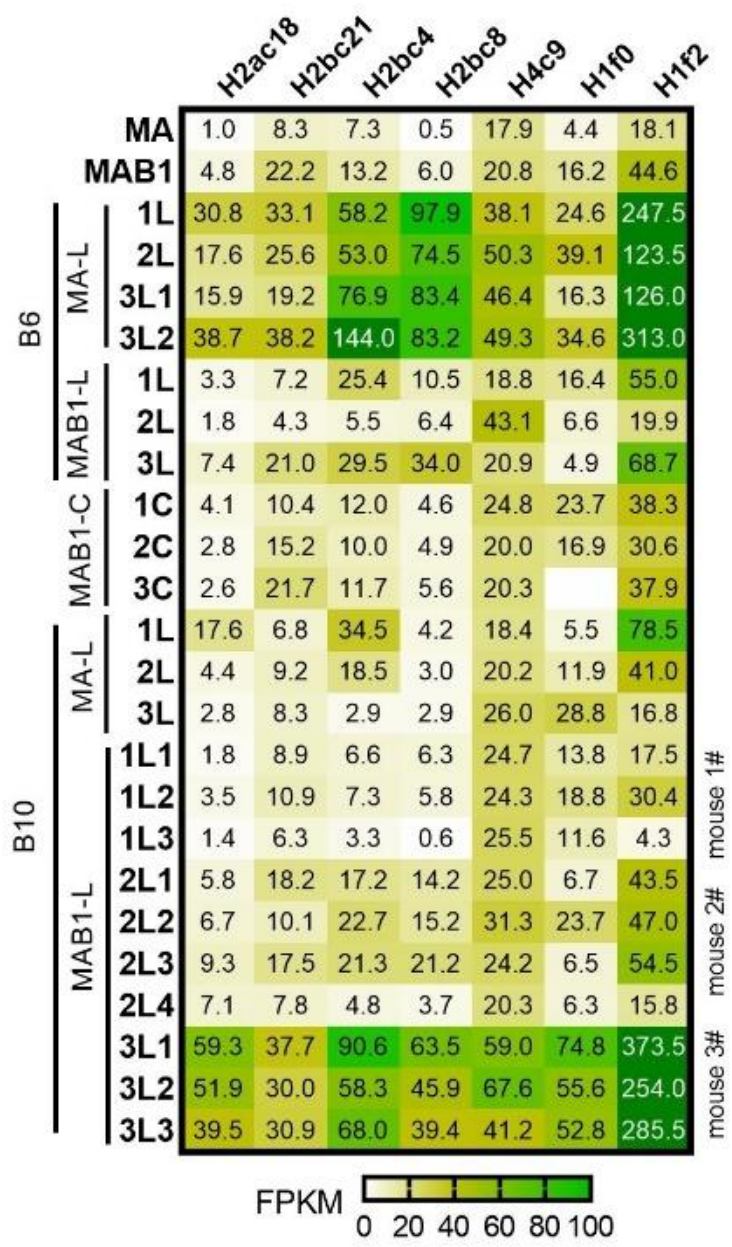

Fig. S4. The expression patterns of histone cluster genes across various samples. A. The expression patterns of HISTs and ZFPs in each comparison group. B. The number of H1 linker genes and H2A/H2B/H3/H4 genes in each comparison group. C. HISTs in DEGs of comparison groups. Low expression, FPKM value <2. Medium expression, 2<FPKM value <20. High expression, FPKM value >20. D. The expression patterns of 7 high-expression HISTs genes in all samples.

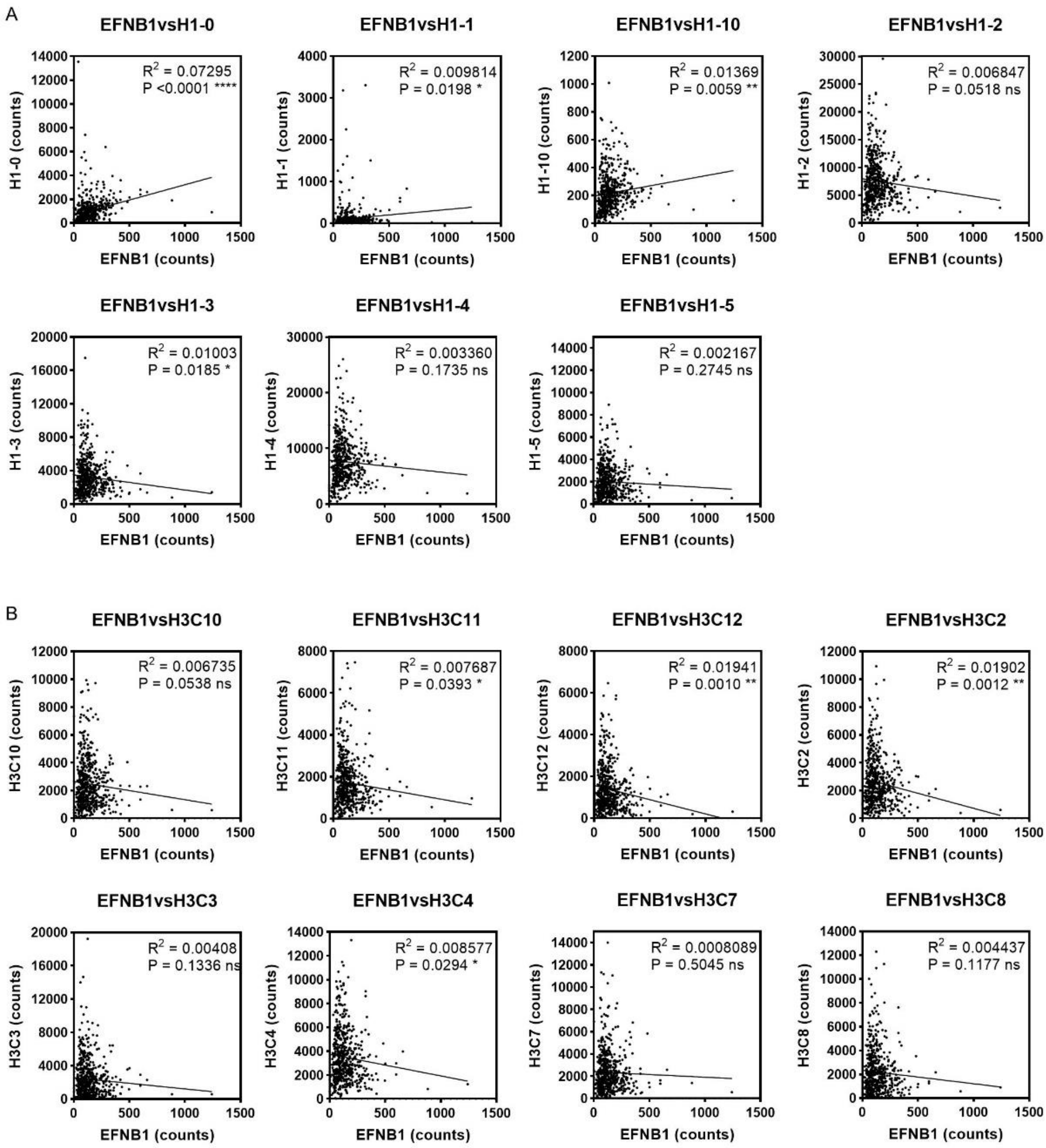

Fig. S5. The expression levels of EFNB1 correlate with those of histone H1 and histone H3. A. The correlation analysis between the expression levels of EFNB1 and those of histone H1 genes. B. The correlation analysis between the expression levels of EFNB1 and those of histone H3. Simple linear regression was used to analysis the statistical differences.

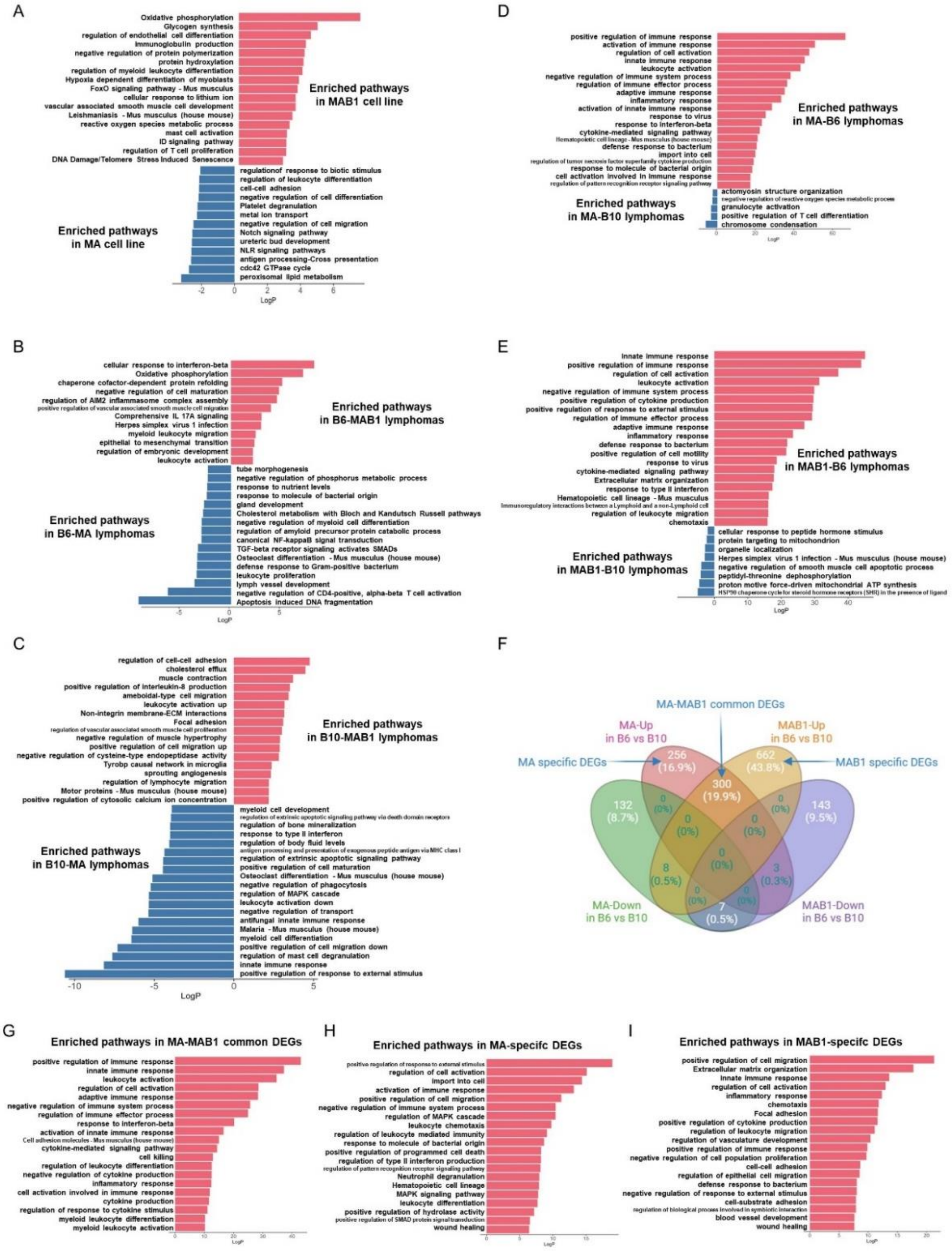

Fig. S6. Enriched pathways of DEGs in each comparison group. A. Enriched pathways for DEGs in MA versus MAB1 cell lines. B. Enriched pathways for DEGs in MA versus MAB1 lymphoma arising from B6 recipient mice. C. Enriched pathways for DEGs in MA versus MAB1 Lymphoma arising from B10 recipient mice. D. Enriched pathways for DEGs in B6-MA versus B10-MA lymphoma. E. Enriched pathways for DEGs in B6-MAB1 versus B10-MAB1 lymphoma. F. DEGs of B6-MA versus B10-MA lymphoma and B6-MA versus B10-MA lymphoma. G-I. Enriched pathways of up-regulated MA-MAB1 common DEGs (G), MA-specific DEGs (H), MAB1-specific DEGs (I).

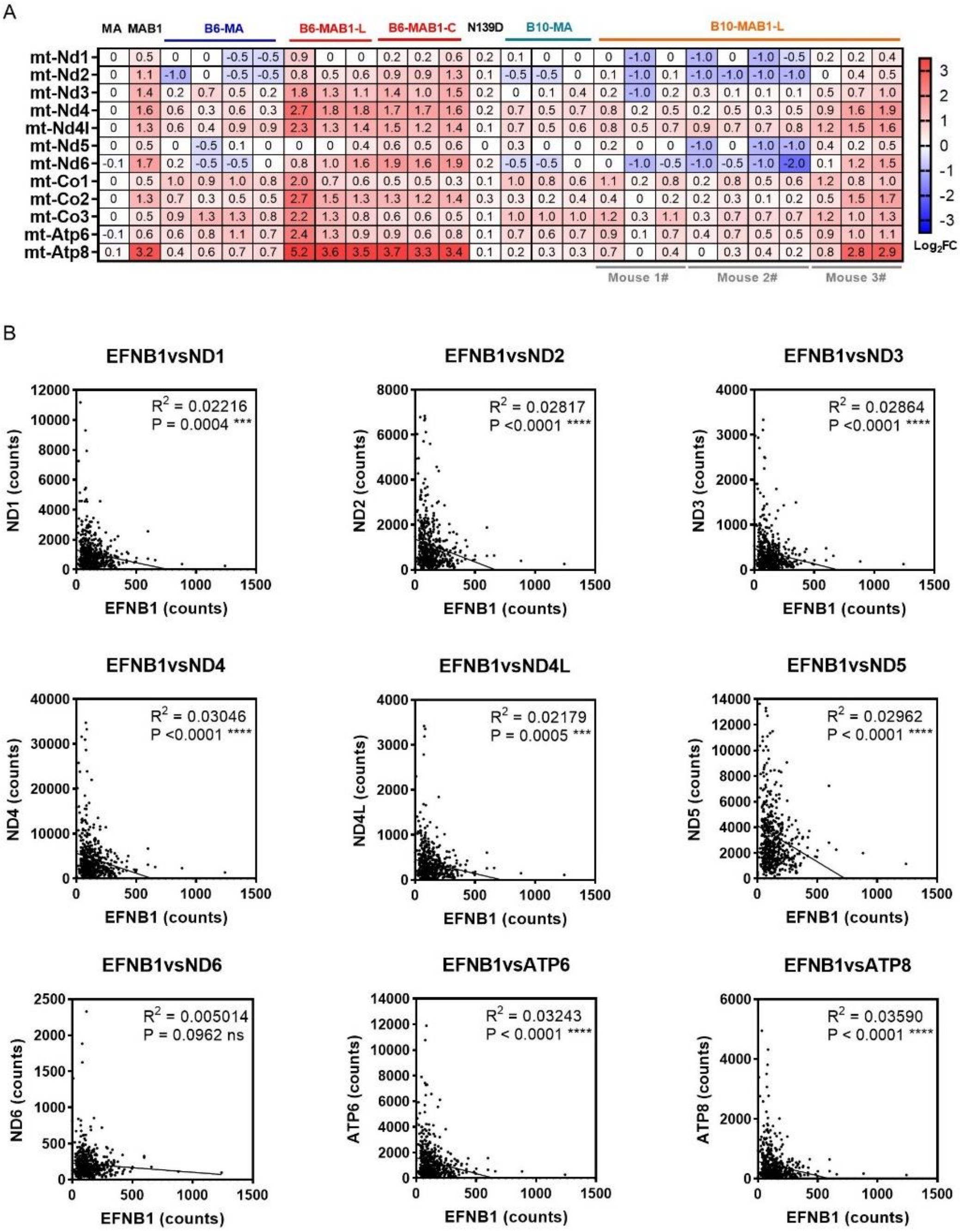

Fig. S7. The expression levels of EFNB1 correlate with those of mitochondrial respiratory chain genes. A. The expression patterns of mitochondrial respiratory chain genes in all samples. All samples were normalized to the MA cell line. B. The correlation analysis between the expression levels of EFNB1 and those of mt-ND genes and mt-ATP. Simple linear regression was used to analysis the statistical differences.

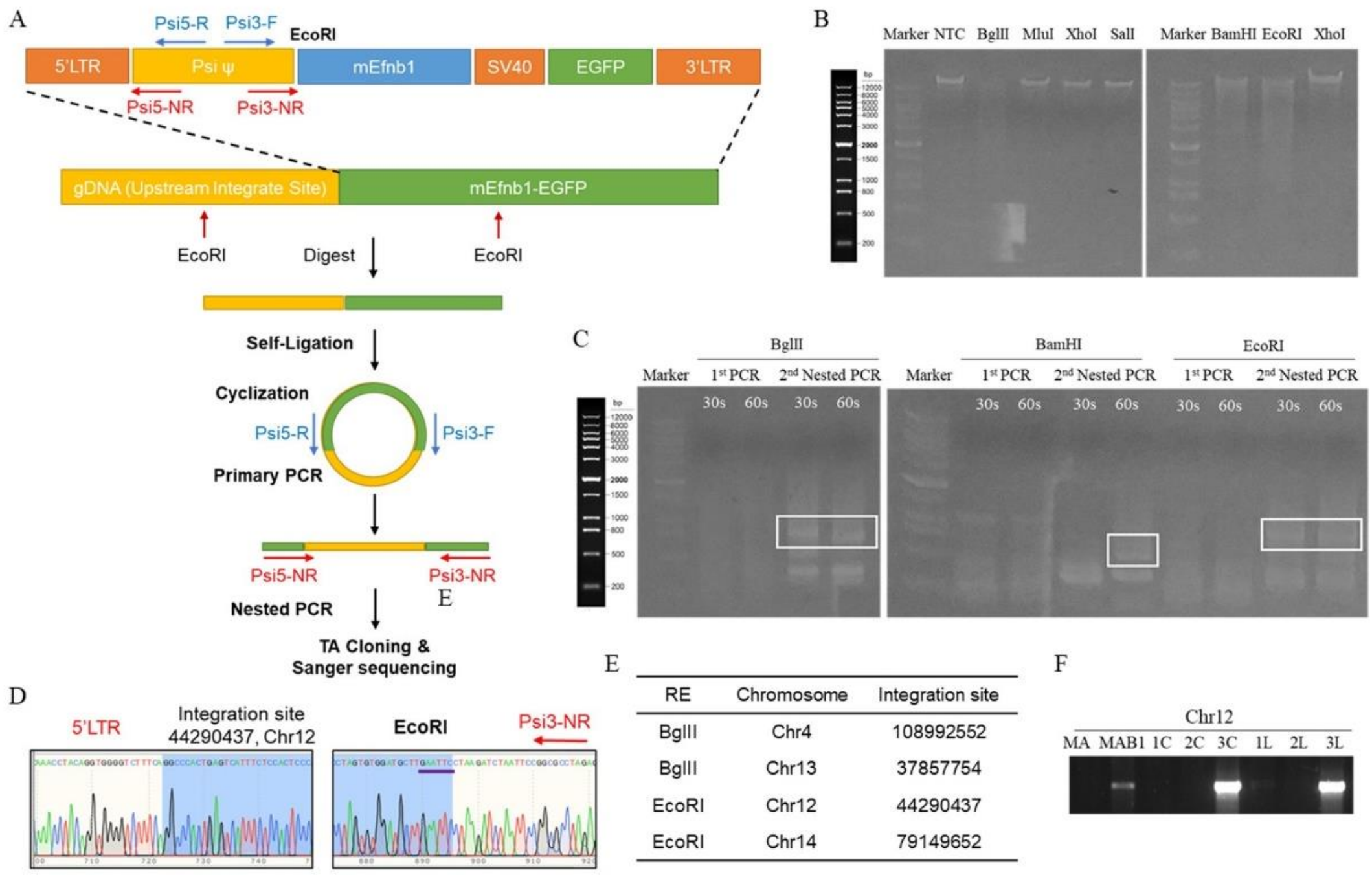

Fig. S8. Retroviral integration site analysis by inverse PCR. A. Overview of retroviral integration site analysis protocol. B. Screening of genome-degradable endonucleases for integration site analysis. C. Electrophoretic analysis of primary and nested PCR amplicons for integration site detection. D. Representative Sanger sequencing results confirming integration sites. E. All integration sites identified by Sanger sequencing. F. Validation of cell strain clones harboring site-specific retroviral integrations

Table S1. Information of syngeneic lymphoma reconstruction experiments

Experiments	Involved Figure	Transplanted cells	Mouse strain	Mouse number	Tumor incidence	Lymphoma name	Established cell strains
1	Figure 1B	MAB1	C57BL/6J	4	4/4	MAB1-11L, -12L, -13L, -14L	
2	Figure 1C	MAB1	C57BL/6J	4	4/4	MAB1-21L, -22L, -23L, -24L	MAB1-Lung (MAB1-22L)
3	Figure 2D	MA	C57BL/6JGpt	5	5/5	MA-1L, -2L, -3L1/2, -4L	MA-2, MA-31, MA-32
3	Figure 2E	MA	C57BL/10JGpt	5	4/5	B10-MA-1L, -2L, -3L, -4L	MAB1-1, MAB1-2, MAB1-3
3	Figure 2F	MAB1	C57BL/6JGpt	5	5/5	MAB1-1L, -2L, -3L, -4L	
3	Figure 2G	MAB1	C57BL/10JGpt	5	4/5	B10-MAB1-1L1/2/3, B10-MAB1-2L1/2/3/4, B10-MA-3L1/2/3, B10-MAB1-4L	
4	Figure 8B	MAB1-1	C57BL/6J	5	3/5	MAB1-1-1L, -2L, -3L	
4	Figure 8C	MAB1-2	C57BL/6J	5	5/5	MAB1-2-1L, -2L, -3L, -4L, -5L	
4	-	MAB1-3	C57BL/6J	5	0/5	Failed to lymphomagenesis	
5	Figure 8D	MAB1-Lung	C57BL/6J	2	2/2	MAB1-Lung-1L, -2L	
6	-	MA 1# clone	C57BL/6J	5	1/3	Failed to lymphomagenesis	
6	-	MA 2# clone	C57BL/6J	5	0/3	Failed to lymphomagenesis	
6	-	MA 3# clone	C57BL/6J	5	0/3	Failed to lymphomagenesis	

Table S2. Primers for SNVs verification and Ig rearrangement analysis

Primer name	Primer sequence
Ttk-gDNA-F	5'-AGTGATGTCTGGTCCTTGGG-3'
Ttk-gDNA-R	5'-CATCCTGCTCAGACATCGGT-3'
Ttk-cDNA-F	5'-CAGCCAGACACAACAAGCAT-3'
Ttk-cDNA-R	5'-CCTGGATGGGGCTGAATTTG-3'
Nckipsd-gDNA-F	5'-GGTGGTGTGCAGAGGTCTTA-3'
Nckipsd-gDNA-R	5'-TTTGCTCCCCATACGATCCA-3'
Nckipsd-cDNA-F	5'-CTCCTCTCGCTGGTAGAAGG-3'
Nckipsd-cDNA-R	5'-CGTAGCTTGTCTCCAGGTGA-3'
Med1-gDNA-F	5'-GTTGTAGGAGCTTCTCGGGA-3'
Med1-gDNA-R	5'-TCCTGGGTTTAGTCCTCAGC-3'
Med1-cDNA-F	5'-AGAATGGACTGGGCTCTCAC-3'
Med1-cDNA-R	5'-CATCACCACACACACAAGGG-3'
Slc39a11-gDNA-F	5'-ATGGACTGTGTGTGGAGGAG-3'
Slc39a11-gDNA-R	5'-AGACTGGACCCCTCTGAGAT-3'
Slc39a11-cDNA-F	5'-GAGGCGGATCTTAGACGGAA-3'
Slc39a11-cDNA-R	5'-TCATCACCACAAATCCCAGGA-3'
Ighv2-2-F	5'-CAGGGTTTCAGAAGAGGGACT-3'
Ighv2-2-R	5'-ATGGCTGTGTCATCAGCTTG-3'
Ighv2-3-F	5'-TGTCAGGGTTTCAGAAGAGGA-3'
Ighv2-3-R	5'-CGTGGCTGTGTCATCAGTTT-3'
Ighv5-2-F	5'-CCAGCACTCTTTGTTTGCAG-3'
Ighv5-2-R	5'-AGGCTGTGTCCTCAGACCTC-3'
Igkv3-1-F	5'-TCAGTCCCCTGGATTTTCTG-3'
Igkv3-1-R	5'-ACCCACTGCCACTAAACCTG-3'
Igkv4-70-F	5'-GGCAGGGAATTTGAGATCAG-3'
Igkv4-70-R	5'-CCAGAAGCCAGTTTGGATGT-3'
Igkv13-85-F	5'-TGACATTGGAATTCATTTGCTT-3'
Igkv13-85-R	5'-TCCACTGCCACTGAATCTTG-3'
Igkv19-93-F	5'-TCAGGAATTTCTGGTCCTTCA-3'
Igkv19-93-R	5'-TGCAATATCTTCAGGCTCCA-3'
